# Using cyclic voltammetry to probe the conformational transition of short elastin-like peptides

**DOI:** 10.1038/s42004-026-01987-8

**Published:** 2026-04-01

**Authors:** Sogol Asaei, Caeden E. Couch, Elena Ising, Luisa R. Parker, Nicholas Sinclair, Julie N. Renner

**Affiliations:** Department of Chemical and Biomolecular Engineering, Case Western Reserve University, Cleveland, OH USA

**Keywords:** Characterization and analytical techniques, Biomaterials - proteins, Peptides

## Abstract

Elastin-like polypeptides (ELPs) are proteins naturally inspired by the hydrophobic domain of tropoelastin. ELPs are the repeating pentapeptide sequences of VPGXG (X can be any amino acid except proline) that undergo inverse transition behavior with temperature and salt stimuli. However, it remains challenging and unclear whether this transition, often accompanied by a conformational change, can be detected for short ELPs which are immobilized on surfaces. In this study, a sensing platform was developed based on the electrochemical activity of tyrosine to show conformational changes of newly designed, tethered, short ELPs with different hydrophobicity on gold working electrodes. Specifically, ELP sequences were modified with an N-terminal cysteine tag to attach to a solid gold surface via a thiol bond and modified with a C-terminal tyrosine tag, which can undergo electrochemical oxidation at the right potential. The ability of ELP conformational changes to modulate the oxidative current and indicate transition behavior was investigated via cyclic voltammetry. Additionally, the transition behavior of the short ELPs in solution was analyzed by UV-visible spectrometry for comparison. Overall, this study explores a distinct method for quantifying and studying the transition behavior of short, engineered peptides on a gold surface.

## Abstract (web summary)

Elastin-like polypeptides (ELPs) exhibit inverse transition behavior with temperature and salt stimuli, often accompanied by a conformational change, but whether this transition can be detected for short ELPs immobilized on surfaces remains underexplored. Here, the authors develop a sensing platform based on the electrochemical activity of tyrosine to show conformational changes of newly designed, tethered, short ELPs with different hydrophobicity on gold working electrodes, probing the effect of conformational changes on the oxidative current using cyclic voltammetry.

## Introduction

Elastin-like polypeptides (ELPs) are stimuli-responsive biopolymers inspired by the sequences of mammalian tropoelastin^1^. ELPs are mostly defined as repetitive pentapeptides consisting of Val-Pro-Gly-X-Gly (VPGXG), where X is a guest residue that can be any amino acid except proline^2,3^. ELPs are considered thermoresponsive proteins that exhibit stimuli-responsive behavior^4^ similar to what is observed in poly (N-isopropylacrylamide) (PNIPAAM), widely recognized as a prominent thermoresponsive polymer^5^. ELPs can be dissolved in an aqueous solution; however, upon increasing the temperature, their solubility changes leading to aggregation at the inverse transition temperature (Tt). They can reversibly return to their soluble state upon lowering the temperature^4,6^.

Tt is related to the lower critical solution temperature (LCST), which is the lowest temperature at which the phase transition occurs. Transition behavior is accompanied by changes in hydration (coordination of water molecules around the ELPs) from structurally ordered water surrounding the hydrophobic moieties of the ELP below the Tt to more disorganized water above the Tt; in other words, ELP structures change from an extended, well-hydrated, and dynamic random coil to a collapsed, less hydrated structure with more β-turns^2,7^. Different environmental stimuli can instigate ELPs to undergo a phase transition, such as solution pH, solution ionic strength, ELP hydrophobicity (controlled via the guest residue on the fourth position in the repeated pentapeptide sequence of ELP), ELP concentration, and ELP length^7,8^. The transition behavior can be precisely engineered; thus, ELPs have found many bioengineering applications, such as therapeutic drug delivery, protein purification, tissue engineering, removal of heavy metals, nano-medicine, bio and chemical sensing, and other biomedical applications^9–11^.

Compared to the extensive research available on long-chain elastin-like polypeptides, studies on short elastin-like peptides consisting of 1–6 pentapeptide repeats remain relatively scarce^7,12–17^. Previous studies have shown that conformational changes can occur even in peptides as short as a single pentapeptide repeat GVG(VPGVG) in solution^17,18^. Reiersen et al.^17^ used circular dichroism to reveal that conformational change is intrinsic to individual pentameric units. This transition is driven by entropy changes and influenced by sequence modifications and non-polar interactions. They observed that β-turn structure, which forms as temperature increases, resulted from interactions within individual molecules rather than between different molecules. In other words, the conformational changes are driven by intramolecular forces, not intermolecular associations in solution. Several studies have investigated temperature-dependent transitions in short elastin-like peptides containing one to three repeats and demonstrated that these transitions arise from intramolecular conformational rearrangements coupled to changes in hydration water^18–20^. Li et al. reported that temperature-dependent conformational changes within individual ELP molecules are sufficient to promote aggregation above the transition temperature. These results support a collective mechanism in which transition behavior arises from coupled single-chain structural rearrangements and changes in peptide hydration, governed by competing peptide–peptide and peptide–water interactions. Further, they showed that a single ELP chain in aqueous solution can adapt a collapsed state over the temperature range of ~290–350 K by atomistic simulations. Their simulations indicate that the transition is initiated by intramolecular interactions of individual ELP molecules, which then promote intermolecular association above the LCST^21^. Therefore, there is evidence transition can occur in short ELP sequences.

Although the stimuli-responsive behavior of ELPs has been widely studied in solution via UV-visible spectroscopy to track changes in opacity, dynamic light scattering to observe shifts in the hydrodynamic radius^22,23^, and circular dichroism to analyze secondary structure^17^, their behavior remains less well understood when ELPs are grafted on a surface. ELPs may exhibit different behavior when surface-bound compared to when they are in solution because their movement is more limited^24^. While a few previous studies have focused on the application of tethered ELPs, such as in electrochemical biosensing^25,26^, gene and drug delivery^27^, cell culture and regenerative medicine^27^, and antibody immobilization^28^, quantification of surface-bound ELP transition behavior is not as robust as ELP free in solution, making transition temperature prediction more difficult. The work on three-armed star ELPs, which demonstrated how structural confinement and micelle formation affect the Tt, provides potential evidence that such restrictions significantly influence the thermal responsiveness of ELPs^29^. Christensen et al.^30^ have observed that fused ELPs experience structural and dynamic restrictions, including reduced flexibility, steric hindrance, and altered hydrodynamic properties due to interactions with fusion proteins, which significantly impact their transition temperature compared to free ELPs. There are some technical approaches that can measure the transition behavior of thermoresponsive polymers attached to solid surfaces, such as quartz crystal microbalance with dissipation (QCM-D), contact angle measurement, electrochemical impedance spectroscopy (EIS), and atomic force microscopy^31^. However, there is a lack of simple techniques sensitive enough to measure the transition of short surface-bound ELPs.

Electrochemical measurements are a promising option to quantify the transition of short ELPs on surfaces because they offer benefits such as exceptional sensitivity, straightforward design, and ease of use^32^. Tryptophan, tyrosine, cysteine, histidine, and methionine are electroactive amino acids where their oxidation is utilized in the analysis of proteins and peptides^33^. In this electrochemical approach, as the protein aggregates and folds the diffusion of electroactive moieties to the surface of the electrode changes, which can be detected by differences in measured current^34^. This technique has been used in oxidative footprinting to study solvent-accessible amino acids in intact, folded proteins^35^. Suprun et al.^36^ investigated the accessibility of electroactive amino acids such as histidine, methionine, and tyrosine to a carbon screen-printed electrode (SPE) surface in phosphate buffer with a pH of 7.2 using peptides similar to amyloid-beta (Aβ) peptide. Another study also observed conformational changes of Aβ peptide upon metal binding by changes in the current via electroactive moieties^37^. Feeney et al. used electrochemical impedance spectroscopy and cyclic voltammetry (CV) to show the improvement of reproducibility of ELP-modified surfaces in their recent publication^38^. Our group electrochemically detected conformational changes of a tyrosine-conjugated, thiol-terminated, gold SPE-bound, EF-hand loop peptide as it binds to a cerium ion for rare earth element sensing^39^. The design platform in our previous work is particularly attractive for detecting conformational changes in other short peptides, such as ELPs.

In this study, we aimed to develop a simple technique that can be used to measure and study surface-bound ELP transition behavior. Since for surface-grafted ELPs the formation of a bulk peptide-rich phase is geometrically constrained, the conformational transition behavior is therefore perhaps better described as a surface-limited conformational transition that may include limited interchain contacts but may not necessarily constitute a classical phase separation. Therefore, when describing the surface transition behavior in this study, we will use the term transition behavior generally, or conformational transition. Our approach consisted of using electrochemistry to detect ELP conformational changes when assembled on a gold SPE. Specifically, a cysteine tag was added at the N-terminus, facilitating binding of ELP to the gold working electrode via a thiol bond, and an electroactive tyrosine was added to the C-terminus. Changes in current as salt or temperature varied were used to detect transition behavior. Herein, we investigated five ELPs with different hydrophobicity by UV-Vis spectroscopy in solution and our distinct electrochemical approach when attached to a surface.

## Results and discussion

### Short ELP design

Elastin-like polypeptides (ELPs) were designed based on the pentapeptide repeat sequence of VPGXG (where X is a guest residue) to investigate the effect of sequence hydrophobicity on the transition behavior. The starting point for these designs was a sequence studied by Reiersen et al.^17^. Their sequence, called peptide J (Ac-GKL(VPGVG)(VPGVG)(VPGVG)ILG-NH_2_)^17^, had a reported transition temperature of 18 °C at pH 7 in a 10 mM phosphate buffer solution. ILELPV3 sequence was inspired by, not identical to, the peptide reported by Reiersen et al. Our ILELPV3 variant differs in several key aspects: (1) an additional glycine at the N-terminus (reported to increase the transition temperature by ~6 °C)^17^, (2) two additional glycines at the C-terminus (expected to further elevate transition temperature), (3) flanking of tyrosine as an electroactive active amino acid, and (4) conjugation of cysteine for thiol binding. These modifications increase hydrophilicity. To confirm the increase in hydrophilicity increased the structural transition temperature measured via circular dichroism (CD) as expected, we performed experiments in similar conditions (explained in Supplementary Note 1) to Reiersen’s report^17^ and observed an elevated transition temperature (Supplementary Fig. 1).

To systematically study the impact of hydrophobicity, ILELPIV2, ILELPI2V, and ILELPI3 were designed by making substitutions to the guest residue (X) within the VPGXG repeat unit of ILELPV3. Specifically, valine (V) was replaced with isoleucine (I), which is more hydrophobic than valine, in one pentamer for ILELPIV2, two pentamers for ILELPI2V, and all three pentamers for ILELPI3. This stepwise increase in hydrophobicity was expected to progressively lower the transition temperature of these sequences. ELPV3 was then designed as a highly hydrophilic peptide, featuring three repeats of the VPGVG sequence, while leucine (L) and isoleucine (I) adjacent to the elastin repeat were removed to decrease the hydrophobicity. Due to its lower hydrophobicity, this sequence was expected to exhibit the highest transition temperature compared to the other designed peptides.

In addition to these core sequence modifications, all ELP sequences were designed with additional structural elements for experimental purposes. A cysteine residue was added at the N-terminus to facilitate thiol binding to a gold working electrode. A tyrosine residue was conjugated at the C-terminus as a redox-active amino acid for electrochemical studies. Additionally, three glycine residues were introduced at the C-terminal as a flexible linker between the ELP sequence and the tyrosine moiety. To improve stability, all ELP sequences were acylated (Ac) at the N-terminus and amidated (NH_2_) at the C-terminus to prevent degradation and enhance peptide stability.

Table 1 summarizes the five designed ELP sequences, each consisting of three repeats of VPGXG with varying guest residues to modify hydrophobicity. The average hydrophilicity was determined using the Hopp & Woods hydrophilicity scale (verified by website: bachem.com), which averages by summing the hydrophilic index of each amino acid in the ELP sequence and dividing by the total number of amino acids^40^. On the last column, we have also calculated the average hydrophilicity based on the different guest residues’ hydrophilicity in the ELP sequence.

**Table 1:** Five designed ELP sequences for this study consist of three repeats of VPGXG with different guest residues to modify the hydrophobicity of the sequence

Peptide name	Molecular weight	Peptide sequence	Average hydrophilicity of full sequence	Average hydrophilicity based on different guest residues
ELPV3	2023.37	Ac-CGKLVPGVGVPGVGVPGVGGGGY-NH_2_	−0.48	a
ILELPV3	2249.69	Ac-CGKLVPGVGVPGVGVPGVGILGGGY-NH_2_	−0.59	−1.5
ILELPIV2	2263.71	Ac-CGKLVPGIGVPGVGVPGVGILGGGY-NH_2_	−0.60	−1.6
ILELPI2V	2277.74	Ac-CGKLVPGIGVPGIGVPGVGILGGGY-NH_2_	−0.61	−1.7
ILELPI3	2291.77	Ac-CGKLVPGIGVPGIGVPGIGILGGGY-NH_2_	−0.62	−1.8

A cysteine residue has been added to facilitate gold binding, and a tyrosine tag is conjugated as a redox-active amino acid to the C-terminal. The average hydrophilicity is calculated by Hopp & Woods’ hydrophilicity scale.

^a^ELPV3 has different flanking amino acids than the other peptides in this study so the average hydrophilicity was not calculated.

### Transition behavior of short ELPs

**Fig. 1:**
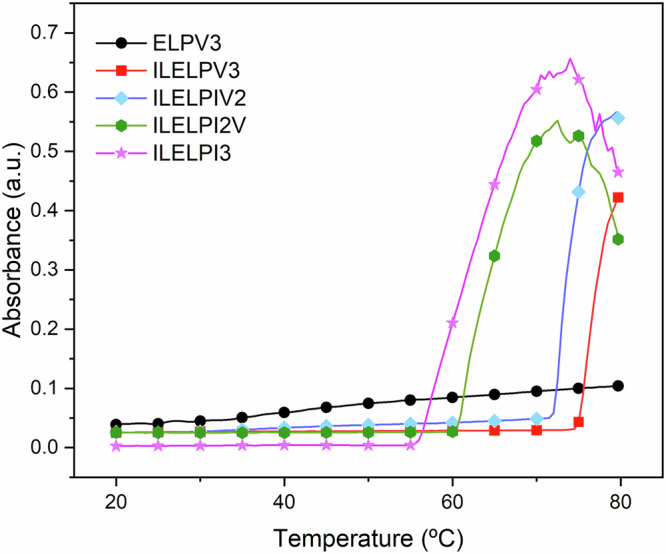
UV–vis heating profiles reveal hydrophobicity-dependent shifts in ELP transition temperature. UV-visible spectroscopy absorbance data for 1 mg/mL peptide solution dissolved in 1 M NaCl as a function of temperature (20–80 °C) with a ramp rate of 0.5 °C/min. These heating profiles show transition temperature decreases as the hydrophobicity of the ELP sequence increases. Lines are added to guide the eye to see the transition behavior. Each data point represents a single absorbance value read at a specific temperature.

UV-visible spectroscopy data were gathered to measure aggregation via absorbance with increasing temperature for short ELPs in solution. These experiments were conducted to understand the impact of temperature and salt on transition behavior in solution and confirm trends were as expected. Designed ELPs at 1 mg/mL and no additional salt did not exhibit any transition behavior within the specified temperature range (20–80 °C) (linear flat lines observed). Therefore, 1 M NaCl was added to all samples. The inverse transition temperature (Tt) was determined as the temperature at which the ELP solution is at 50% of its maximum turbidity^41,42^. Figure 1, which includes only the heating profile from 20 °C to 80 °C, for clarity, reveals that ELPV3 does not display transition behavior in 1 M NaCl conditions. The main factor resulting in different transition temperatures is the different guest residues with varying hydrophobicity. As expected, increasing the hydrophobicity of the peptide sequence results in a lower transition temperature with a Tt of ~76 °C, 74 °C, 64 °C, and 63 °C for ILELPV3, ILELPIV2, ILELPI2V, and ILELPI3, respectively.

All peptide samples underwent a cooling ramp from 80 °C to 20 °C following heating. Supplementary Fig. 2 shows both the heating and cooling profiles for each peptide sequence and displays the reversible behavior of the thermally induced transition of ILELPV3, ILELPIV2, ILELPI2V, and ILELPI3 upon cooling. Hysteresis in ELPs is understood to result from non-equilibrium states and can be influenced by several factors, including chain length, heating rate, and the strength of peptide–peptide interactions. Strong interactions between peptides can delay disaggregation during thermal cycling, causing the ELPs to remain aggregated even after the temperature falls below the expected transition point. This behavior mirrors mechanisms observed in other low-complexity protein systems, where similar structural features lead to hysteresis due to hindered molecular disentanglement^43,44^.

Supplementary Fig. 3 shows the fully reversible stimulus-responsive behavior of ILELPIV2, representative of designed ELPs, as it reverts completely back to the initial absorbance after cooling down. Collectively, these experiments confirm that the designed peptides are capable of reversible phase transition behavior and behave as expected with decreasing transition temperature as hydrophobicity increases.

### Characterization of short ELP adsorption on gold via QCM-D

QCM-D data provide insights into mass adsorption on a solid surface and the viscoelastic properties of the assembled layer. Supplementary Fig. 4 shows the frequency shift versus time for the peptides in this study. The frequency drops correlated to mass loading on the gold surface are remarkably similar for all ELPs, indicating similar mass adsorption for all ELP-functionalized samples. The Sauerbery equation was applied to calculate the mass loading on the surface because the viscoelasticity is low enough to consider the samples as thin and rigid layers^39^. The loaded mass on the surface for all designed ELPs in this study is estimated to be 300 ± 20 (ng/cm^2^) and the thickness of the peptide on the surface is around 3 nm.

Despite using a relatively low peptide concentration of 10 mg/mL compared to the concentration used to functionalize the electrochemical sensors, the ELPs rapidly covered the gold surface of the QCM-D gold-coated sensors. Complete coverage was achieved in less than 2 min. This rapid adsorption behavior suggests that the concentration of 1 mg/mL used for electrochemical experiments, with an incubation time of 10 min, is sufficient to ensure complete coverage and saturation of the electrode surface. Supplementary Fig. 5 demonstrates frequency shifts vs. the time when 1 mg/mL solution of ILELPI3 (as an example of designed ELPs) flowed over the gold sensor. QCM-D data with 1 mg/mL showing similar loading after rinsing confirms that the higher concentration does not result in a significantly different loading due to multilayers.

### Atomic force microscopy of assembled peptide layers on gold

AFM was employed to image the topography of short ELPs immobilized on gold surfaces. A bare gold-coated QCM-D sensor without any peptide served as a control surface for comparison. The AFM height images, presented in Supplementary Fig. 6, reveal remarkably similar topographies for all designed ELPs. Our observations indicate that immobilization of the peptides did not significantly alter the surface morphology of the gold surface and formed thin monolayers. These results complement our QCM-D results, providing visual confirmation of the consistent ELP layer formation.

### Electrochemical experiments to assess structural changes of surface-bound ELPs in response to salt (NaCl)

Cyclic voltammetry (CV) is an electrochemical technique that reveals current changes as electrochemical reactions take place when an appropriate potential is applied to a working electrode^45^. CV is used to analyze electroactive species and is widely applied in investigating redox processes in fields such as biochemistry and macromolecular chemistry^36,46^. In this study, we leveraged the electrochemical properties of tyrosine, an electroactive amino acid, to monitor the conformational changes resulting from the transition behavior of elastin-like polypeptides (ELPs). When peptides containing a tyrosine tag were immobilized on gold screen-printed working electrodes a tyrosine oxidation peak appeared at around ~0.55 V during CV with an electrolyte solution of 0.1 M potassium phosphate and 0.05 M NaCl^39^. Supplementary Fig. 7 presents a representative CV plot from an electrode functionalized with ILELPV3. In this study, peak height was defined as the vertical distance between the oxidation peak and the baseline, as calculated by Nova Software.

All ELPs in this study were functionalized on a gold screen-printed electrode, and CV was performed in varying concentrations of NaCl to measure the peak heights associated with tyrosine oxidation. All CV experiments have been done at 22 ± 2 °C otherwise stated. CV experiments were repeated 3–7 times for each peptide with a desired salt concentration in a potassium phosphate buffer solution, and the results were averaged across all replicates, each performed on a single sensor. The peak heights were normalized to the highest average peak height observed for each peptide. Normalized peak heights were plotted against salt concentration and then modeled with a four-parameter logistic model in Minitab to identify the inflection point (the minimum constraint for the curves was set to 0). The logistic model is defined in Eq. (1):

1 $$Y={{{\rm{q}}}}1+\frac{{{{\rm{q}}}}2-{{{\rm{q}}}}1}{1+{e}^{\frac{x-{{{\rm{q}}}}3}{{{{\rm{q}}}}4}}}$$

Where Y is the normalized peak height, q1 is the low plateau (normalized peak heights at high salt concentration), q2 is the high plateau (normalized peak heights at low salt concentration), q3 is the midpoint (inflection point), q4 is the width of the curve, and x is the salt concentration^47,48^. The use of a logistic model was chosen as an empirical model. Empirical sigmoidal fitting and inflection-point analysis are widely used to characterize elastin-like polypeptide transitions. Dreher et al.^49^ reported the critical micelle concentration determined from the inflection point of a sigmoidal fit to fluorescence data describing an ELP transition. De Haas et al.^50^ determined the transition temperature of ELP by fitting normalized turbidity data with a sigmoidal function and defining the transition temperature as the inflection point of the fit. Similar sigmoidal analyses are commonly used to characterize ELP transition points under varying environmental conditions^50–53^.

**Fig. 2:**
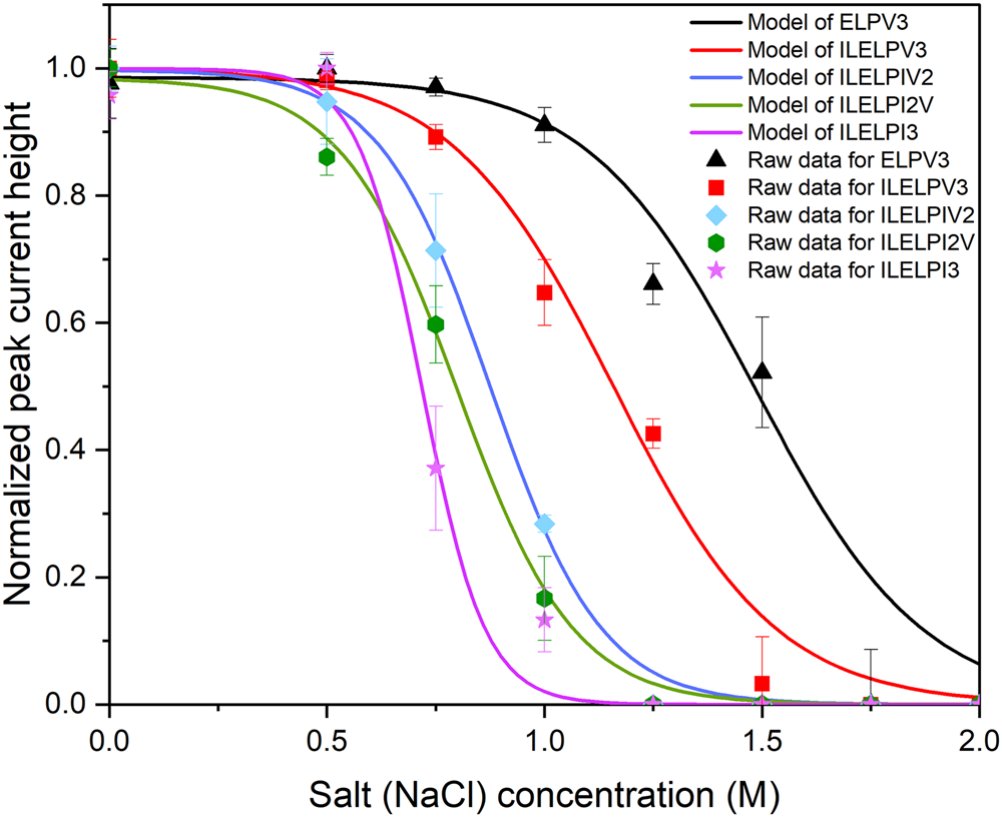
Normalized CV peak current varies with NaCl concentration across surface-tethered ELP variants. Normalized peak current height versus salt (NaCl) concentration obtained by CV for ELPV3 (black triangles show data average, black line shows model), ILELPV3 (red squares show data average, red line shows model), ILELPIV2 (blue rhombus show data average, blue line shows model), ILELPI2V (green parallelograms show data average, green line shows model), and ILELPI3 (purple stars show data average, purple line shows model). The normalized peak current height of CV data was averaged for different numbers of repeats  (Supplementary Table 1 reports the precise number of replicates per data point shown in the plot with each repeat being done on a single sensor) and modeled via Eq. (1). CV was performed between 0 to 0.7 V vs. a silver pseudo-reference electrode with a scan rate of 50 mV/s. Error bars represent standard error.

The resulting plot, illustrated in Fig. 2, shows that as NaCl concentration increases in the electrolyte solution, relative peak heights decrease, indicating that ELPs respond to salt in such a way as to inhibit tyrosine oxidation. This reduction in peak height as salt is added may be attributed to the conformational changes of the ELP layer under high-salt conditions, which likely impedes the diffusion of the redox species (tyrosine). Speculatively, transitioned tethered ELP chains undergo salt-induced structural rearrangements that either restrict mobility and/or cover the electrode surface. As a result, fewer tyrosine moieties are able to make productive electrochemical contact with the electrode, which manifests as a decrease in current (peak height). It is unknown if these structural changes manifest in only a single molecule or as the interaction of multiple molecules on the surface (see *Surface-bound Short ELP Transition Behavior Discussion*). Regardless, this behavior is consistent with what was reported in previous literature with longer ELP molecules, which also used NaCl as a stimulus for conformational changes^26^.

In addition, as ELP hydrophobicity increases (ILELPI3 being the most hydrophobic), less salt is needed to elicit the response. This behavior is consistent with transition behavior for ELPs in solution, where there is an expected decrease in ELP transition temperature as salt concentration increases, with more hydrophobic ELPs having lower transition points^54,55^. Table 2 summarizes the inflection point (M) resulting from data fitting to Eq. (1), providing quantitative insights into the salt sensitivity of each ELP variant. ILELPI3, the most hydrophobic variant, transitions at ~0.72 M NaCl, while ILELPI2V, ILELPIV2 and ILELPV3 require higher salt concentrations of 0.80 M and, 0.88 M, and 1.16 M, respectively.

**Table 2:** Salt concentration at which the designed ELPs transitioned

Peptide name	Salt concentration (M) ± standard error at inflection point
ILELPV3	1.16 ± 0.05
ILELPIV2	0.88 ± 0.02
ILELPI2V	0.80 ± 0.02
ILELPI3	0.72 ± 0.03

Inflection point (M) is identified as the transition point on CV plots, which are modeled with Eq. (1) ± standard error associated with the model.

We also investigated ELPV3, which did not transition in our solution-based experiments, as a control. As salt is added to the ELPV3-functionalized electrode, a similar decrease in peak current is observed, but at a higher salt concentration than in other ELP samples. To confirm if the behavior was associated with transition behavior, another peptide called LanM1Y in our previous study^39^, which does not have an ELP sequence and is not known to transition, was analyzed for comparison (see Supplementary Fig. 8). The curves overlap, suggesting ELPV3 might not transition under these conditions. These control experiments imply there is an upper limit of NaCl concentration for this electrochemical technique when it is applied to measuring surface-bound transition behavior. We assumed the gradual decrease in peak current height observed for all ELPs, even for non-ELP, is attributed to gold chloride formation on the electrode surface.

To corroborate that gold-bound peptides are displaying transition behavior, we performed some supporting experiments. Another salt, sodium sulfate (Na_2_SO_4_), was selected as a stimulus with examples of ELPV3 and ILELPI3 (ELPV3, expected to either not transition or have a very low transition based on our previous experiment with NaCl and ILELPI3 as an ELP that displays transition behavior). Sodium sulfate is considered a kosmotrope in the Hofmeister series^56^. Sho et al. reported the temperature-dependent aggregation behavior of some ELPs via different hydrophobicity exposed to 11 sodium salts. Sodium sulfate induced a transition of ELPs at around six times lower in concentration than sodium chloride^54^. Therefore, in our study, cyclic voltammetry was performed at a lower concentration of sodium sulfate (between 0 and 1 M) than sodium chloride (between 0.05 and 2 M). Peak current height data were normalized similarly to what we did for sodium chloride experiments. Supplementary Fig. 9 shows normalized peak current height versus sodium sulfate concentration. As shown in Supplementary Fig. 9, less salt is needed to induce a reduction in current for surfaces modified with ILELPI3, as expected compared to NaCl. In addition, ELPV3 likely did not undergo transition. These are single-repetition tests for qualitative confirmation rather than quantitative. The data are in good agreement with the effect of sodium sulfate on the transition behavior of ELPs reported previously^54^.

In addition to this, another technique was used to evaluate if reversible behavior was present. The Fe(CN)₆³⁻/⁴⁻ redox couple is often used to check how much of the electrode surface is accessible. If the surface is blocked, the redox peaks get smaller or disappear, which relates to a smaller electrochemically active surface area^57^. In this technique, peptide-modified electrodes were first exposed to a high salt environment (1 M NaCl solution), and CV was performed (red curve in Supplementary Fig. 10 shows these CV results); subsequently, the salt was rinsed off to remove the salt stimulus before performing cyclic voltammetry a second time (blue curve in Supplementary Fig. 10 shows these CV results). Another peptide-functionalized sensor was exposed to the iron ferricyanide redox probe without salt, as shown by the green curve in Supplementary Fig. 10. If the ELPs’ stimulus-responsive behavior is reversible, the blue and green curve scans should overlap. The close overlap between the blue and green CV traces confirms that the electrochemical behavior returns to its original state after stimulus removal. The difference between the green and red curves confirms that the transition does cause a notable change in the CV behavior, with lower current observed when NaCl is present. The red curves in the presence of salt also show that in the case of ELPV3 transition does not occur, as expected. Similarly, for ILELPV3, 1 M NaCl does not induce a full transition as it is near the inflection point (see Fig. 2). The other ELP samples have a more drastic change when NaCl is present. A control done on bare gold indicates that the simple presence of salt does not cause this change (Supplementary Fig. 10f). Overall, these findings support the conclusion that the ELPs exhibit reversible conformational transitions at the electrode interface, consistent with our UV-Vis data showing solution-phase reversibility.

### Electrochemical experiments to assess structural changes of surface-bound ELPs in response to temperature

Temperature was also investigated as another stimulus to confirm that the surface-bound ELPs were demonstrating transition behavior. Cyclic voltammetry was performed within the same potential window used for the salt stimuli experiments in the presence of 0.4 M NaCl. The concentration of 0.4 M NaCl was chosen since it can depress the transition temperature to be within experimental range, while ELPs are not yet transitioned based on Fig. 2. The electrode’s response is governed by the movement of electroactive species between the electrode surface and the bulk solution^44^. As temperature increases, the current also increases, primarily due to enhanced diffusion of electroactive species. Higher temperatures also reduce the viscosity of the solution, allowing both the electrolyte ions and the peptide chains (including tyrosine residues) to move more freely^58^. This increased molecular mobility facilitates more frequent interactions between the redox-active tyrosine groups and the electrode surface. Thus, the electrode’s current response increases with temperature due to faster mass transport and enhanced reaction kinetics^44,59^.

**Fig. 3:**
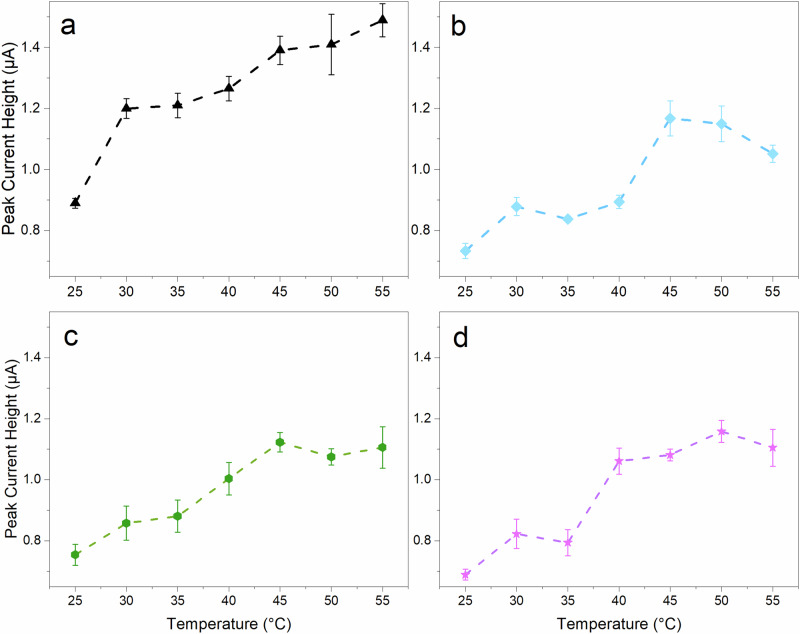
CV peak current height versus temperature shows distinct thermal transitions across ELP sequences. Peak current height versus temperature plot of electrodes functionalized with **a** ELPV3, **b** ILELPIV2, **c** ILELPI2V, **d** ILELPI3. Supplementary Table 3 reports the precise number of replicates per data point shown in the plots (each repeat was done on a single sensor). Cyclic voltammetry was performed from 0 to 0.7 V vs. a silver pseudo-reference electrode with a scan rate of 50 mV/s. Error bars represent ± standard errors. Segments are to connect the reader’s eye to the next consecutive temperature. Example CVs for ILELPI3 are provided in Supplementary Fig. 13.

Figure 3 shows current height versus temperature for ELPV3, ILELPIV2, ILELPI2V, and ILELPI3. ELPV3 is not expected to transition, and peak current increases with increasing temperature. In contrast, for ELPs expected to transition, as temperature increases, the peak height increases but slows and eventually plateaus. This plateau indicates that the ELP undergoes transition: once the tethered ELPs undergo structural transition onto the electrode surface, they limit electron accessibility, and further temperature increases no longer significantly increase the peak current within the range investigated. Speculatively, the temperature-driven enhancement of current may be counteracted by the reduced surface accessibility of ELPs, resulting in a true plateau rather than a temporary one. This slowing down trend happens at lower temperatures for more hydrophobic ELPs. Statistical analysis was performed for peptides shown in Fig. 3 to determine if the plateau did occur earlier for more hydrophobic peptides. Supplementary Table 2 presents all statistical analyses. ILELPIV2 and ILELPV3 were analyzed using one-way ANOVA followed by Tukey’s post hoc test. ILELPI2V did not meet normality assumptions and was analyzed using the Kruskal–Wallis test followed by Dunn’s post hoc comparisons. ILELPI3 exhibited unequal variances and was analyzed using Welch’s one-way ANOVA followed by a Games–Howell post hoc test. The analysis reveals that ILELPIV2 has a plateau occurring around 45 °C (with 40 and 45 °C being statistically different). ILELPI2V, while harder to detect due to high error, indicates that 35 and 45 °C are significantly different, with transition perhaps occurring in that range. ILELPI3 has a distinctly lower plateau than ILELPIV2 with 35 and 40 °C being significantly different. Supplementary Fig. 11 shows ILELPV3 might undergo a transition in the range of 45–50 °C, which is above the temperature found for the more hydrophobic peptide ILELPIV2, as expected. ELPV3 did not show a clear plateau over the tested temperature range; therefore, no transition temperature was assigned.

### Surface-bound short ELP transition behavior discussion

In the previous sections, we presented data demonstrating that surface-bound short ELPs are capable of typical stimuli-responsive behavior in solution and presented an electrochemical technique that is capable of measuring transition behavior when tethered to a surface. Comparison between solution and surface behavior is challenging since the transition temperature depends on ELP concentration^25^, and the exact concentration of the peptide on the surface while similar among the tethered ELPs in this study, is unknown. However, generally, we observe that the transition temperature is lower on the surface than in solution. In our system, the peptides are tethered to a gold surface, which introduces additional effects that are not present in solution in that restricted mobility can significantly increase effective local concentration. To help understand this result, a rough estimate of surface concentration was attempted. ELPs are highly hydrated, where water molecules persist within the structure to satisfy backbone hydrogen bonding requirements. This substantial hydration volume, ~60% by mass, suggests that the net peptide concentration at the surface, excluding the hydration shell, is lower than the apparent concentration derived from QCM-D data which measure hydrated mass^60^. We detail these estimates in Supplementary Eqs. (1)–(9) (explained by Supplementary Notes 2 and 3^61^) and show that the ELP concentration on the surface is likely to correspond to several hundred-fold higher local concentration than that used in solution turbidity measurements. In addition, the surface concentrations are likely sufficient to promote interchain interactions. While it seems the short ELPs could achieve sufficient proximity to support multichain interactions, we cannot distinguish between intramolecular and intermolecular interactions in this study and further investigation is required. Future studies might use our electrochemical technique in combination with other tools such as surface plasmon resonance (SPR) to understand the behavior as a function of surface concentration, highlighting the utility of the different method.

**Fig. 4:**
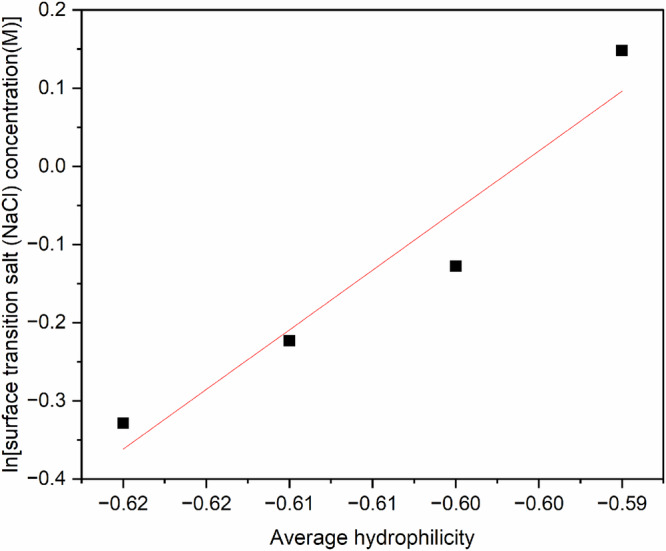
Peptide hydrophilicity has a linear relationship with the natural logarithm of the surface transition salt concentration. Linear fit of the natural logarithm of the surface transition salt (NaCl) concentration versus the hydrophilicity of peptides calculated in Table 1. The red line represents the linear regression model shown in Eq. (2). Each data point shows the average hydrophilicity of each peptide at its surface transition salt (NaCl) concentration.

Previous studies have proposed models to predict ELP transition temperatures in solution; however, no established models exist for surface-immobilized ELPs. Our data suggest a linear relationship exists between the average hydrophobicity of ELPs and the natural logarithm of the surface transition salt concentration (Fig. 4). Equation (2) details the model that best fits our data, which is obtained by Minitab linear regression curve fitting:

2 $${{{\rm{LN}}}}({{{\rm{S}}}})=(9.1\pm 1.8)+(15.3\pm 3.0){{{\rm{H}}}}$$

Where S is the surface transition salt concentration (in molar, M), and H is the average hydrophilicity of the designed ELPs shown in Table 1. The intercept, slope, and R-squared are shown in Supplementary Fig. 12, and Supplementary Table 4 summarizes ANOVA results for this linear regression. The slope and intercept are shown with the standard error obtained from the regression. The p-value of the linear regression was less than the chosen α value, representing that the regression is statistically significant. While limited to this specific molecular weight of ELP at room temperature, this relationship could serve as a valuable tool for predicting the transition behavior of other tethered ELP sequences with known hydrophobicity.

## Conclusion

In this study, we employed electrochemical approaches to characterize the conformational changes of surface-bound elastin-like polypeptides (ELPs). Each ELP sequence was engineered with a cysteine tag to facilitate gold surface binding and a tyrosine residue that enabled electrochemical detection of conformational changes. The electrochemical activity of tyrosine served as a biosensing mechanism to monitor conformational changes.

UV-Vis spectroscopy was used to characterize the transition behavior of the peptides in solution and confirm their reversible thermal responsiveness. ILELPV3, ILELPIV2, ILELPI2V, and ILELPI3 exhibited reversible transitions between 20 °C and 80 °C in 1 M NaCl, while ELPV3 did not transition under these conditions. The transition temperature decreased as the average hydrophobicity of the peptide increased, as expected.

Electrochemical experiments were then performed to investigate whether the transition behavior could be detected when the peptides were immobilized on a gold surface. We observed that the peak current height resulting from tyrosine oxidation decreased with increasing salt concentration, indicating conformational changes of the tethered ELPs. To model the salt-dependent transition behavior of surface-bound ELPs, we applied a logistic growth model. This model revealed the specific salt concentrations at which transitions occurred for each peptide: ILELPV3, ILELPIV2, ILELPI2V, and ILELPI3 transitioned at 0.72 M, 0.80 M, 0.88 M, and 1.16 M NaCl, respectively. We also demonstrated that the salt-induced conformational changes were reversible. Finally, we found that a significant linear relationship exists between the natural logarithm of the surface transition salt (NaCl) concentration and the average hydrophilicity of ELPs.

Together, these results establish electrochemistry as a robust platform for quantifying conformational transitions of surface-tethered ELPs, offering potential for predictive modeling in surface-based elastin biotechnologies. Such predictive modeling enables the rational design of ELP sequences with tailored transition behaviors at specific salt concentrations or temperatures. These designed systems could be applied in drug delivery, where transitions at physiological temperature or salinity release of therapeutics, or in cell culture applications that exploit responsive surface properties. Furthermore, they hold promise in sensing technologies, for example, by detecting and capturing specific ions, changing hydrophilicity/water organization and detecting subsequent conformational changes.

## Methods and materials

### Chemicals and reagents

Hydrogen peroxide solution (30% in water) and dimethyl sulfoxide (DMSO) (>99.9%) were obtained from Sigma-Aldrich. Potassium phosphate monobasic was acquired from VWR, and potassium phosphate dibasic (≥98%) was purchased from EMD Millipore. Ammonium hydroxide solution (28–30%) was obtained from J.T. Baker, and Dot Scientific supplied sodium hydroxide. Potassium hexacyanoferrate (III) (≥99.0%) and potassium hexacyanoferrate (II) (98.5–102.0%) were obtained from Sigma-Aldrich. Deionized water (DI) used for QCM-D experiments was DNAse- and RNAse-free and sourced by Invitrogen. Milli-Q (MQ) water was produced using a Millipore purification system (resistivity = 18.2 MΩ·cm). High-purity nitrogen gas (HPNG) (99.99%) was procured from Airgas. Sodium sulfate anhydrous (99%) was purchased from Fisher Scientific. Sodium dodecyl sulfate (SDS, 99%) used for QCM-D module cleaning was purchased from Hoefer. Peptides designed for the study were purchased from Genscript (https://www.genscript.com/) with a purity of ≥95.0% in lyophilized form. The purity of these peptides was evaluated by GenScript using high-performance liquid chromatography (HPLC), with accompanying analysis reports disclosing the most suitable solvent for the peptide based on its hydrophobicity. All peptides were obtained from Genscript in 1 mg gross weight vials and used without further modification.

### Peptide solution preparation

All peptides (ELPV3–ILELPI3) were initially dissolved in 100 μL of DMSO due to their hydrophobic nature and then diluted with 900 μL of MQ water to prepare stock solutions (e.g., 1 mg of peptide in 100 μL DMSO and 900 μL MQ water), resulting in final solutions containing ~10% DMSO by volume and a concentration of 1 mg/mL. All ELP solutions were stored at –20 °C, as recommended by GenScript.

### UV-visible spectroscopy

UV-visible absorption spectroscopy data were taken using an Agilent Cary 3500 Peltier UV-Vis Spectrometer, which has been equipped with a temperature controller. The absorbance was measured in an ultra-micro rectangular quartz cell with a 10 mm path length and 70 µL fill volume sealed with a Teflon stopper. Samples of 1 mg/mL peptide solution were prepared with 1 M NaCl solution. Sample absorbance was measured at 420 nm while the temperature was increased from 20 °C to 80 °C at a rate of 0.5 °C min^−1^ and back down from 80 °C to 20 °C with the same rate. The transition temperature (Tt) was defined as the temperature at which the ELP solution is at 50% of its maximum turbidity^41,42^. After testing, one peptide (ILELPIV2) was selected to screen for full reversibility by storing at −20 °C. The ILELPIV2 sample was frozen and thawed and then placed in the UV-Vis spectrometer to measure absorbance at 420 nm at a temperature of 20 °C for a hold of 2 minutes. These results were compared to the data from the original testing of the peptide solution corresponding to 20 °C.

### Quartz crystal microbalance with dissipation (QCM-D)

The QCM-D instrument (QSense Explorer, Biolin Scientific) specifications and cleaning processes for the flow module (QFM 401) and gold-coated crystal sensors (QSX 301, 5 MHz, Biolin Scientific) have been thoroughly explained in our previous work^39^. Briefly, frequency shifts (∆f) and dissipation changes (∆D) with time as peptides adsorbed on the gold surface were recorded by QCM-D. Dissipation shifts are used to assess viscoelasticity, whereas frequency shifts are associated with mass absorption. Generally, a negative shift in frequency indicates an increase in adsorbed mass. After the baseline was established, where stability was maintained for 10 min, the peptide solution (10 µg/mL) was introduced into the flow module at a rate of 150 µL/min while the temperature was maintained at 18 °C. The QSense software can calculate the mass loaded on the surface with predefined models based on the frequency drop^62^. The Sauerbery equation can be used for loaded mass calculations when the layer is thin and rigid without significant viscoelasticity and was used in this study^39^.

### Atomic force microscopy

Surfaces with adsorbed peptide monolayers were evaluated using the Bruker Dimension 3100 Veeco Digital Instruments atomic force microscopy (AFM) in tapping mode with a FESPA-V2 AFM probe (probe specifications: nominal spring constant of 2.8 N/m and resonance frequency of 75 kHz; tip specifications: Sb-doped Si, and nominal radius of 8 nm) and a scan rate of 0.256 Hz. The images were captured at 500 × 500 nm^2^. AFM scans were taken on sensors with immobilized peptide after running QCM-D experiments and letting them dry at room temperature. Scans were conducted in a chamber under ambient temperature and atmospheric pressure. The NanoScope Analysis Program V 1.50 was used to process and analyze the AFM data.

### Electrochemical experiments

Cyclic voltammetry (CV) measurements were performed using a PGSTAT101 Metrohm potentiostat (Metrohm Autolab, Netherlands) with Nova software. Screen-printed electrodes (SPE, 220 BT) with a gold working electrode (WE), silver pseudo reference electrode (RE), and gold counter electrode (CE) were used for these experiments. As explained in our previous work^39^, an oxidation peak is anticipated to be observed in peptide-functionalized sensors containing tyrosine around 0.55–0.65 V vs. an Ag pseudo-reference electrode at a pH of around 7. To functionalize gold working electrodes with peptide, the sensor was incubated for 10 min with a 10 µL drop of 1 mg/mL peptide solution. The sensors were rinsed with MQ water to remove loosely bound peptides and dried with high-purity nitrogen gas. Then, CV experiments were conducted with 70 mL of 0.1 M potassium phosphate buffer at pH 7.4 and different concentrations of salt (NaCl (0.05–2 M) or Na_2_SO_4_ (0–1 M)-depending on the experiments) to investigate salt as a stimulus for conformational changes. Each screen-printed electrode was used once due to the irreversible oxidation of tyrosine at the conditions of this study.

To analyze the impact of temperature, sensors were immersed in 0.1 M potassium phosphate at pH 7.4 + 0.4 M NaCl buffer solution at a defined temperature ranging from 25 °C to 55 °C. Tubes with the buffer solution were placed in a temperature-controlled dry bath. The real temperature was set by measuring the temperature of the buffer solution, not what the dry bath showed. Cyclic voltammetry was performed from 0 to 0.7 V vs. Ag pseudo-reference at a scan rate of 50 mV s^−1^.

To test the reversibility of the stimulus-responsive behavior, CV scans were performed from –0.2 to 0.6 V vs. an Ag pseudo-reference electrode using two separate screen-printed electrodes (SPEs) at a scan rate of 100 mV s^−1^. On one of the sensors, a CV was taken on immobilized ELP in the absence of salt. On another sensor, a CV was taken in the presence of salt (expected to observe transition and conformational changes) and then a second CV on that same sensor was taken subsequent to rinsing of the salt solution. Buffer consisted of 5 mM equimolar mixture of potassium ferrocyanide (K₄(Fe(CN)₆) and potassium ferricyanide (K₃(Fe(CN)₆) in 0.1 M KCl as the redox probe and 1 M NaCl was added to monitor the effect of salt as a stimulus.

### Statistical analysis

Minitab Version 22.2.1 was used for all statistical analyses. For statistical tests, a significance threshold of α = 0.05 was selected. Nonlinear curve fitting was done on electrochemical data, analyzing the impact of salt addition using the logistic growth model, which is explained in the section containing Fig. 2. For the current response data at different temperatures (Fig. 3), normality was examined via the Anderson-Darling (AD) test and constant variance was examined via Levene’s test. If data were normal and had equal variance, a one-way ANOVA assuming equal variance was performed to see if the factor of temperature had a significant effect on current. If temperature was found to be a significant factor, Tukey’s Honestly Significant Difference (HSD) post hoc test was used to determine which temperature groups differed significantly. For peptides with non-normal data in Fig. 3, a Kruskal–Wallis test was performed and if temperature was found to be a significant factor, a Dunn’s post hoc test was conducted. For peptides that did not have equal variance across different temperatures in Fig. 3, ANOVA was performed assuming unequal variance, and if temperature was found to be a significant factor, the Games–Howell post hoc test was employed. Data are presented as mean ± standard error unless otherwise specified in the figure. Sample sizes (replicates) were based on prior laboratory experience and are described in figure captions as well as SI tables.

## Supplementary information

Supplementary information

Reporting Checklist For Life Sciences Articles

## Data Availability

Data found in the main text of this manuscript can be found at: 10.7910/DVN/3D26NF.

## References

[1] Foster, J. A., Bruenger, E., Gray, W. R. & Sandberg, L. B. Isolation and amino acid sequences of tropoelastin peptides. *J. Biol. Chem.* **248**, 2876–2879 (1973). 4697396

[2] Urry, D. W. Entropic elastic processes in protein mechanisms. I. Elastic structure due to an inverse temperature transition and elasticity due to internal chain dynamics. *J. protein Chem.* **7**, 1–34 (1988). 3076447 10.1007/BF01025411

[3] Gagner, J. E., Kim, W. & Chaikof, E. L. Designing protein-based biomaterials for medical applications. *Acta Biomater.* **10**, 1542–1557 (2014). 24121196 10.1016/j.actbio.2013.10.001 PMC3960372

[4] Li, B., Alonso, D. O. & Daggett, V. The molecular basis for the inverse temperature transition of elastin. *J. Mol. Biol.* **305**, 581–592 (2001). 11152614 10.1006/jmbi.2000.4306

[5] Doberenz, F., Zeng, K., Willems, C., Zhang, K. & Groth, T. Thermoresponsive polymers and their biomedical application in tissue engineering–a review. *J. Mater. Chem. B* **8**, 607–628 (2020). 31939978 10.1039/c9tb02052g

[6] Chilkoti, A., Christensen, T. & MacKay, J. A. Stimulus responsive elastin biopolymers: applications in medicine and biotechnology. *Curr. Opin. Chem. Biol.* **10**, 652–657 (2006). 17055770 10.1016/j.cbpa.2006.10.010 PMC3732176

[7] Nuhn, H. & Klok, H.-A. Secondary structure formation and LCST behavior of short elastin-like peptides. *Biomacromolecules*. **9**, 2755–2763 (2008). 18754687 10.1021/bm800784y

[8] Urry, D. W. Molecular machines: how motion and other functions of living organisms can result from reversible chemical changes. *Angew. Chem. Int. Ed. Engl.* **32**, 819–841 (1993).

[9] Kowalczyk, T., Hnatuszko-Konka, K., Gerszberg, A. & Kononowicz, A. K. Elastin-like polypeptides as a promising family of genetically-engineered protein based polymers. *World J. Microbiol. Biotechnol.* **30**, 2141–2152 (2014). 24699809 10.1007/s11274-014-1649-5 PMC4072924

[10] Wang, E. E. Development of Stimuli-Responsive Elastin-like Polypeptide-based Nanocomposite Biomaterials. (University of California, Berkeley and University of California, San Francisco, CA, 2012).

[11] Despanie, J., Dhandhukia, J. P., Hamm-Alvarez, S. F. & MacKay, J. A. Elastin-like polypeptides: Therapeutic applications for an emerging class of nanomedicines. *J. Controlled Rel.* **240**, 93–108 (2016). 10.1016/j.jconrel.2015.11.010 PMC5767577 26578439

[12] Cook, W. J., Einspahr, H., Trapane, T. L., Urry, D. W. & Bugg, C. E. Crystal structure and conformation of the cyclic trimer of a repeat pentapeptide of elastin, cyclo-(L-valyl-L-prolylglycyl-L-valylglycyl) 3. *J. Am. Chem. Soc.* **102**, 5502–5505 (1980).

[13] Pechar, M. et al. Thermoresponsive Self-Assembly of Short Elastin-Like Polypentapeptides and Their Poly (ethylene glycol) Derivatives. *Macromol. Biosci.* **7**, 56–69 (2007). 17238231 10.1002/mabi.200600196

[14] Flamia, R., Lanza, G., Salvi, A. M., Castle, J. E. & Tamburro, A. M. Conformational study and hydrogen bonds detection on elastin-related polypeptides using X-ray photoelectron spectroscopy. *Biomacromolecules* **6**, 1299–1309 (2005). 15877345 10.1021/bm049290s

[15] Yao, X. & Hong, M. Structure distribution in an elastin-mimetic peptide (VPGVG) 3 investigated by solid-state NMR. *J. Am. Chem. Soc.* **126**, 4199–4210 (2004). 15053609 10.1021/ja036686n

[16] Schreiner, E. et al. Folding and unfolding of an elastinlike oligopeptide:“inverse temperature transition,” reentrance, and hydrogen-bond dynamics. *Phys. Rev. Lett.* **92**, 148101 (2004). 15089575 10.1103/PhysRevLett.92.148101

[17] Reiersen, H., Clarke, A. R. & Rees, A. R. Short elastin-like peptides exhibit the same temperature-induced structural transitions as elastin polymers: implications for protein engineering. *J. Mol. Biol.* **283**, 255–264 (1998). 9761688 10.1006/jmbi.1998.2067

[18] Rousseau, R., Schreiner, E., Kohlmeyer, A. & Marx, D. Temperature-dependent conformational transitions and hydrogen-bond dynamics of the elastin-like octapeptide GVG (VPGVG): a molecular-dynamics study. *Biophys. J.* **86**, 1393–1407 (2004). 14990469 10.1016/S0006-3495(04)74210-1 PMC1303977

[19] Nicolini, C., Ravindra, R., Ludolph, B. & Winter, R. Characterization of the temperature-and pressure-induced inverse and reentrant transition of the minimum elastin-like polypeptide GVG (VPGVG) by DSC, PPC, CD, and FT-IR spectroscopy. *Biophys. J.* **86**, 1385–1392 (2004). 14990468 10.1016/S0006-3495(04)74209-5 PMC1303976

[20] Krukau, A., Brovchenko, I. & Geiger, A. Temperature-induced conformational transition of a model elastin-like peptide GVG (VPGVG) 3 in water. *Biomacromolecules* **8**, 2196–2202 (2007). 17567170 10.1021/bm070233j

[21] Li, N. K., Quiroz, F. G., Hall, C. K., Chilkoti, A. & Yingling, Y. G. Molecular description of the LCST behavior of an elastin-like polypeptide. *Biomacromolecules* **15**, 3522–3530 (2014). 25142785 10.1021/bm500658w

[22] Kurzbach, D. et al. Hydration layer coupling and cooperativity in phase behavior of stimulus responsive peptide polymers. *J. Am. Chem. Soc.* **135**, 11299–11308 (2013). 23822733 10.1021/ja4047872 PMC4167343

[23] MacEwan, S. R. & Chilkoti, A. Applications of elastin-like polypeptides in drug delivery. *J. Controlled Rel.* **190**, 314–330 (2014). 10.1016/j.jconrel.2014.06.028 PMC4167344 24979207

[24] Schweigerdt, A., Heinen, S., Stöbener, D. D. & Weinhart, M. Grafting density-dependent phase transition mechanism of thermoresponsive poly (glycidyl ether) brushes: a comprehensive QCM-D study. *Langmuir* **37**, 7087–7096 (2021). 34077209 10.1021/acs.langmuir.1c00695

[25] Su, Z., Kim, C. & Renner, J. N. Quantification of the effects of hydrophobicity and mass loading on the effective coverage of surface-immobilized elastin-like peptides. *Biochem. Eng. J.* **168**, 107933 (2021).

[26] Morales, M. A., Paiva, W. A., Marvin, L., Balog, E. R. M. & Halpern, J. M. Electrochemical characterization of the stimuli-response of surface-immobilized elastin-like polymers. *Soft Matter* **15**, 9640–9646 (2019). 31670364 10.1039/c9sm01681c

[27] Dai, M. et al. Engineered protein polymer-gold nanoparticle hybrid materials for small molecule delivery. *J. Nanomed. Nanotechnol.* **7**, 356 (2016). 10.4172/2157-7439.1000356 PMC4828936 27081576

[28] Alvisi, N. et al. Self-assembly of elastin-like polypeptide brushes on silica surfaces and nanoparticles. *Biomacromolecules* **22**, 1966–1979 (2021). 33871996 10.1021/acs.biomac.1c00067 PMC8154268

[29] Ghoorchian, A., Cole, J. T. & Holland, N. B. Thermoreversible micelle formation using a three-armed star elastin-like polypeptide. *Macromolecules* **43**, 4340–4345 (2010).

[30] Christensen, T., Hassouneh, W., Trabbic-Carlson, K. & Chilkoti, A. Predicting transition temperatures of elastin-like polypeptide fusion proteins. *Biomacromolecules* **14**, 1514–1519 (2013). 23565607 10.1021/bm400167h PMC3667497

[31] Pramounmat, N. *Study of Elastin-Like Polypeptides Grafted on Electrode Surfaces* (Case Western Reserve University, 2022).

[32] Vanova, V. et al. Peptide-based electrochemical biosensors utilized for protein detection. *Biosens. Bioelectron.* **180**, 113087 (2021). 33662844 10.1016/j.bios.2021.113087

[33] Moulaee, K. & Neri, G. Electrochemical amino acid sensing: a review on challenges and achievements. *Biosensors* **11**, 502 (2021). 34940259 10.3390/bios11120502 PMC8699811

[34] Vestergaard, M. D. et al. A rapid label-free electrochemical detection and kinetic study of Alzheimer’s amyloid beta aggregation. *J. Am. Chem. Soc.* **127**, 11892–11893 (2005). 16117499 10.1021/ja052522q

[35] Roeser, J., Permentier, H. P., Bruins, A. P. & Bischoff, R. Electrochemical oxidation and cleavage of tyrosine-and tryptophan-containing tripeptides. *Anal. Chem.* **82**, 7556–7565 (2010). 20726506 10.1021/ac101086w

[36] Suprun, E. V. et al. Direct electrochemical oxidation of amyloid-β peptides via tyrosine, histidine, and methionine residues. *Electrochem. Commun.* **65**, 53–56 (2016).

[37] Suprun, E. V. et al. Tyrosine based electrochemical analysis of amyloid-β fragment (1-16) binding to metal (II) ions. *Electrochim. Acta* **179**, 93–99 (2015).

[38] Feeney, S. et al. Reproducibly modified elastin-like polymer gold electrode surfaces. *ACS Meas. Sci. Au* **5**, 520–528 (2025). 10.1021/acsmeasuresciau.5c00033 PMC12371586 40861911

[39] Asaei, S., Verma, G., Sinclair, N. S. & Renner, J. N. Electrochemical biosensing of cerium with a tyrosine-functionalized EF-hand loop peptide. *AIChE J.* **70**, e18620 (2024).

[40] Hopp, T. P. & Woods, K. R. A computer program for predicting protein antigenic determinants. *Mol. Immunol.* **20**, 483–489 (1983). 6191210 10.1016/0161-5890(83)90029-9

[41] kyteKyte, J. & Doolittle, R. F. A simple method for displaying the hydropathic character of a protein. *J. Mol. Biol.* **157**, 105–132 (1982). 7108955 10.1016/0022-2836(82)90515-0

[42] Na, K. et al. Smart” biopolymer for a reversible stimuli-responsive platform in cell-based biochips. *Langmuir* **24**, 4917–4923 (2008). 18348578 10.1021/la702796y

[43] Garcia Quiroz, F. et al. Intrinsically disordered proteins access a range of hysteretic phase separation behaviors. *Sci. Adv.* **5**, eaax5177 (2019). 31667345 10.1126/sciadv.aax5177 PMC6799979

[44] Pramounmat, N. et al. Grafting of short elastin-like peptides using an electric field. *Sci. Rep.* **12**, 1–13 (2022). 36333395 10.1038/s41598-022-21672-9 PMC9636273

[45] Marken, F., Neudeck, A. & Bond, A. M. Cyclic voltammetry. In *Electroanalytical Methods: Guide to Experiments and Applications* (ed. Scholz, F.), 57–106 (Springer Berlin Heidelberg, Berlin, Heidelberg, 2010).

[46] Dinu, A. & Apetrei, C. Quantification of tyrosine in pharmaceuticals with the new biosensor based on laccase-modified polypyrrole polymeric thin film. *Polymers* **14**, 441 (2022). 35160431 10.3390/polym14030441 PMC8839761

[47] Harris, T. M., Devkota, J. P., Khanna, V., Eranki, P. L. & Landis, A. E. Logistic growth curve modeling of US energy production and consumption. *Renew. Sustain. Energy Rev.* **96**, 46–57 (2018).

[48] Kucharavy, D. & De Guio, R. Application of logistic growth curve. *Proc. Eng.* **131**, 280–290 (2015).

[49] Dreher, M. R. et al. Temperature triggered self-assembly of polypeptides into multivalent spherical micelles. *J. Am. Chem. Soc.* **130**, 687–694 (2008). 18085778 10.1021/ja0764862 PMC2855373

[50] de Haas, R. J., Ganar, K. A., Deshpande, S. & de Vries, R. pH-responsive elastin-like polypeptide designer condensates. *ACS Appl. Mater. Interfaces* **15**, 45336–45344 (2023). 37707425 10.1021/acsami.3c11314 PMC10540133

[51] Ribeiro, A., Arias, F. J., Reguera, J., Alonso, M. & Rodríguez-Cabello, J. C. Influence of the amino-acid sequence on the inverse temperature transition of elastin-like polymers. *Biophys. J.* **97**, 312–320 (2009). 19580769 10.1016/j.bpj.2009.03.030 PMC2711379

[52] Frey, W., Meyer, D. E. & Chilkoti, A. Thermodynamically reversible addressing of a stimuli responsive fusion protein onto a patterned surface template. *Langmuir* **19**, 1641–1653 (2003).

[53] Tang, J. D., Caliari, S. R. & Lampe, K. J. Temperature-dependent complex coacervation of engineered elastin-like polypeptide and hyaluronic acid polyelectrolytes. *Biomacromolecules* **19**, 3925–3935 (2018). 30185029 10.1021/acs.biomac.8b00837

[54] Cho, Y. et al. Effects of Hofmeister anions on the phase transition temperature of elastin-like polypeptides. * J. Phys. Chem. B* **112**, 13765–13771 (2008). 18842018 10.1021/jp8062977 PMC3475179

[55] Reguera, J., Urry, D. W., Parker, T. M., McPherson, D. T. & Rodríguez-Cabello, J. C. Effect of NaCl on the exothermic and endothermic components of the inverse temperature transition of a model elastin-like polymer. *Biomacromolecules*. **8**, 354–358 (2007). 17291058 10.1021/bm060936l

[56] Kunz, W., Nostro, P. L. & Ninham, B. W. The present state of affairs with Hofmeister effects. *Curr. Opin. Colloid interface Sci.* **9**, 1–18 (2004).

[57] Hostert, J. D. et al. Self-assembly and rearrangement of a polyproline II helix peptide on gold. *Langmuir* **37**, 6115–6122 (2021). 33974431 10.1021/acs.langmuir.0c03583

[58] Xia, C., Kang, W., Wang, J. & Wang, W. Temperature dependence of internal friction of peptides. * J. Phys. Chem. B* **125**, 2821–2832 (2021). 33689339 10.1021/acs.jpcb.0c09056

[59] Elgrishi, N. et al. A practical beginner’s guide to cyclic voltammetry. *J. Chem. Educ.* **95**, 197–206 (2018).

[60] Urry, D. W., Trapane, T. & Prasad, K. Phase-structure transitions of the elastin polypentapeptide–water system within the framework of composition–temperature studies. *Biopolymers* **24**, 2345–2356 (1985). 4092092 10.1002/bip.360241212

[61] Root, D. D., Yadavalli, V. K., Forbes, J. G. & Wang, K. Coiled-coil nanomechanics and uncoiling and unfolding of the superhelix and α-helices of myosin. *Biophys. J.* **90**, 2852–2866 (2006). 16439474 10.1529/biophysj.105.071597 PMC1414572

[62] Dixon, M. C. Quartz crystal microbalance with dissipation monitoring: enabling real-time characterization of biological materials and their interactions. *J. Biomol. Tech.* **19**, 151 (2008). 19137101 PMC2563918

