# Supplementary material for: Using cyclic voltammetry to probe the conformational transition of short elastin-like peptides
Source: Commun Chem. 2026 Apr 1;9:259. doi: 10.1038/s42004-026-01987-8 (PMC13421663; doi:10.1038/s42004-026-01987-8)
Supplement: Supplementary file 1 — Supplementary information [file 42004_2026_1987_MOESM1_ESM.pdf]

**Using cyclic voltammetry to probe the conformational transition of short elastin-like peptides**

**Authors:** Sogol Asaei[1], Caeden E. Couch[1], Elena Ising[1§], Luisa R. Parker[1§], Nicholas Sinclair[1*] and Julie N. Renner[1*]

[1]Department of Chemical and Biomolecular Engineering, Case Western Reserve University, Cleveland, Ohio, United States

[*]Corresponding author

Julie N. Renner: Julie.Renner@case.edu

Nicholas Sinclair: nicholas.sinclair@case.edu

[§]Authors who contributed equally

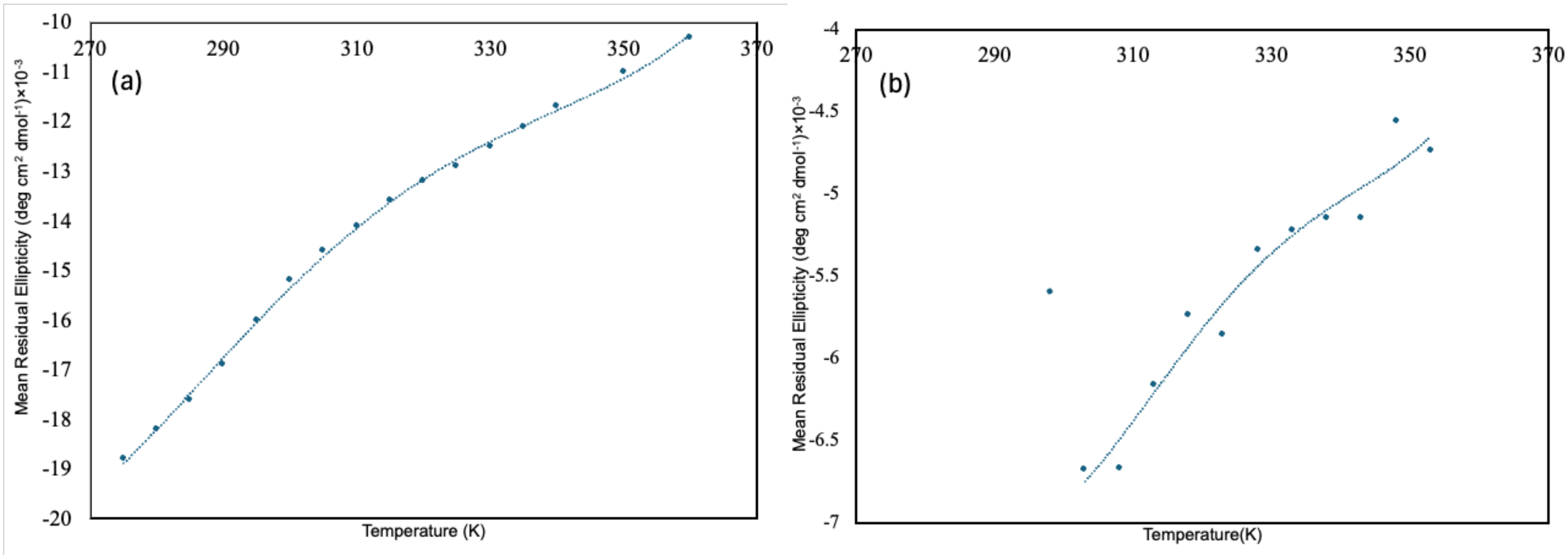


Supplementary Figure 1. CD spectra of 20 µM ILELPV3 in 10 mM phosphate buffer (pH 7), at temperatures between 298 K and 358 K. Scans were processed from 190 nm up to 260 nm, and the average of 5 runs on one sample was subtracted from the baseline and smoothed using the Sabitzky-Golay algorithm in Spectra Manager 2 software. a) Peptide J (data points from Reiersen's article were plotted by visual approximation) and b) ILELPV3 CD data at 199 nm (transition temperature was determined by visual approximation). Peptide J transition temperature has been reported as 18 °C, ILELPV3 transition temperature is around 47 °C. Each data point in part b) represents the average of 5 scans on a single solution, in an experiment performed once. The dotted blue line represents polynomial fitting to guide the eye.

Supplementary Note 1:

**CD Methods**

A Jasco J-815 CD spectropolarimeter, with a Peltier temperature controller and ~300 µL single cuvette holder with 0.1 cm cell length, was used for CD experiments. Spectra Manager2 software was employed for data collection and used to analyze peptide secondary structure in solution. Nitrogen gas was purged into the instrument for 20 minutes before running experiments. Spectra were collected at different temperatures (298 K to 353 K with an increment of 5 °C) using 300 µL of 20 µM peptide solution in 10 mM phosphate buffer (pH 7). The spectral range covered wavelengths

from 190 to 260 nm, with a data pitch of 0.5 nm, a bandwidth of 1.00 nm, and a scanning rate of 50 nm per minute. CD data are represented as the average of 5 repeats on a single solution.

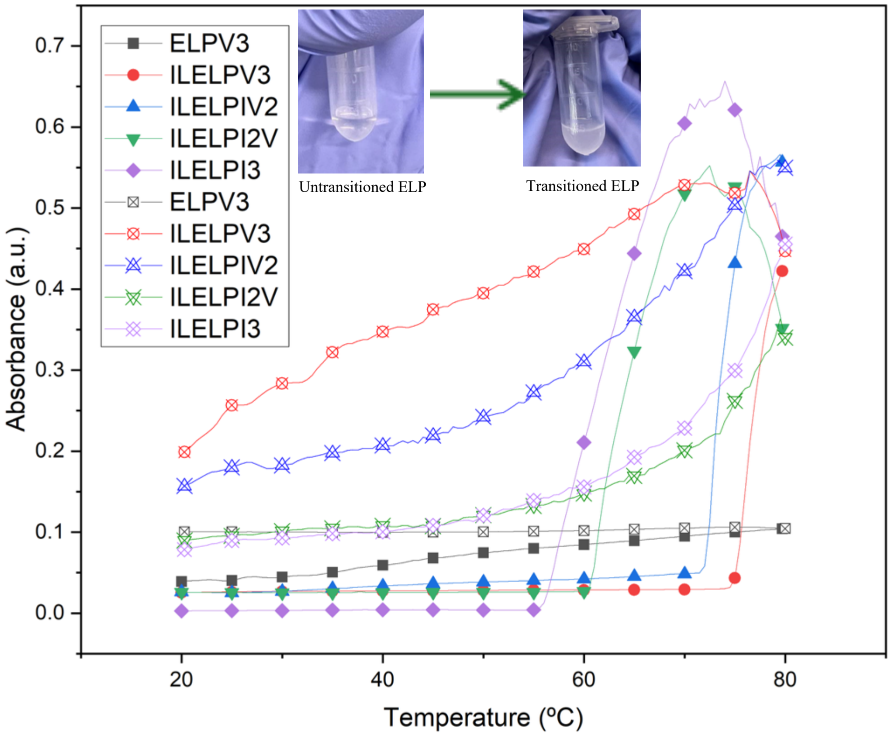


Supplementary Figure 2. UV-visible spectroscopy absorbance data for 1 mg/mL peptide solutions dissolved in 1 M NaCl as a function of temperature (20-80 ºC) with a ramp rate of 0.5 °C/min. These heating profiles show transition temperature decreases as the hydrophobicity of the peptide increases. The cooling profiles (represented in hollow shapes) show the reversibility of transition behavior by decreasing the temperature from 80 °C to 20 °C. Lines are added to guide the eye to see the transition behavior. Each data point represents a single absorbance read at a specific temperature.

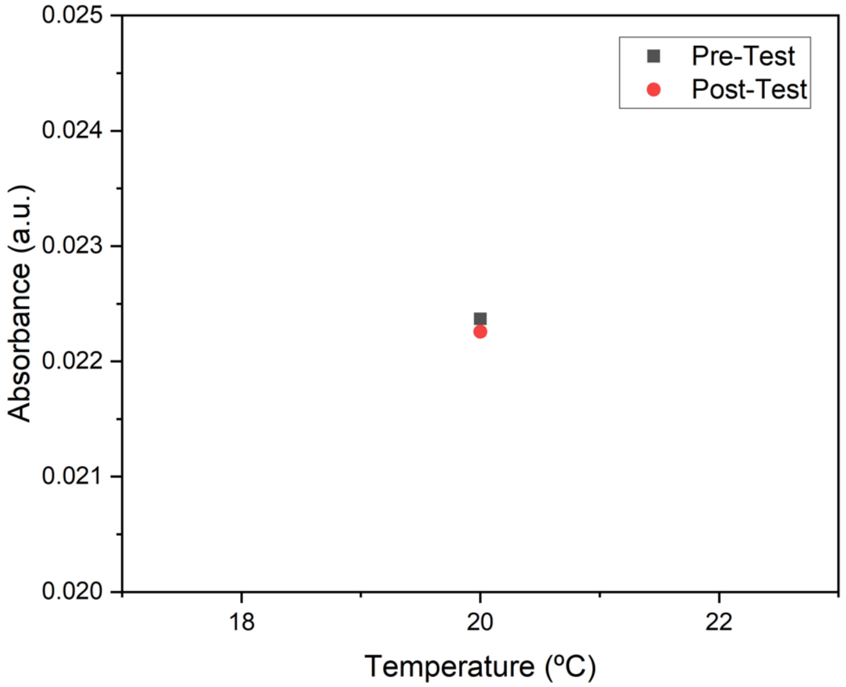


Supplementary Figure 3. UV-visible spectroscopy absorbance data for 1 mg/mL ILELPIV2 solution dissolved in 1 M NaCl before running UV-Vis experiments and after being frozen and thawed post-testing. Data shows reversible stimulus-responsive behavior. The pre-test data point represents the first absorbance read at 20ºC for the up-temperature ramp and the post-test data point represents the last absorbance read at 20ºC for the down-temperature ramp.

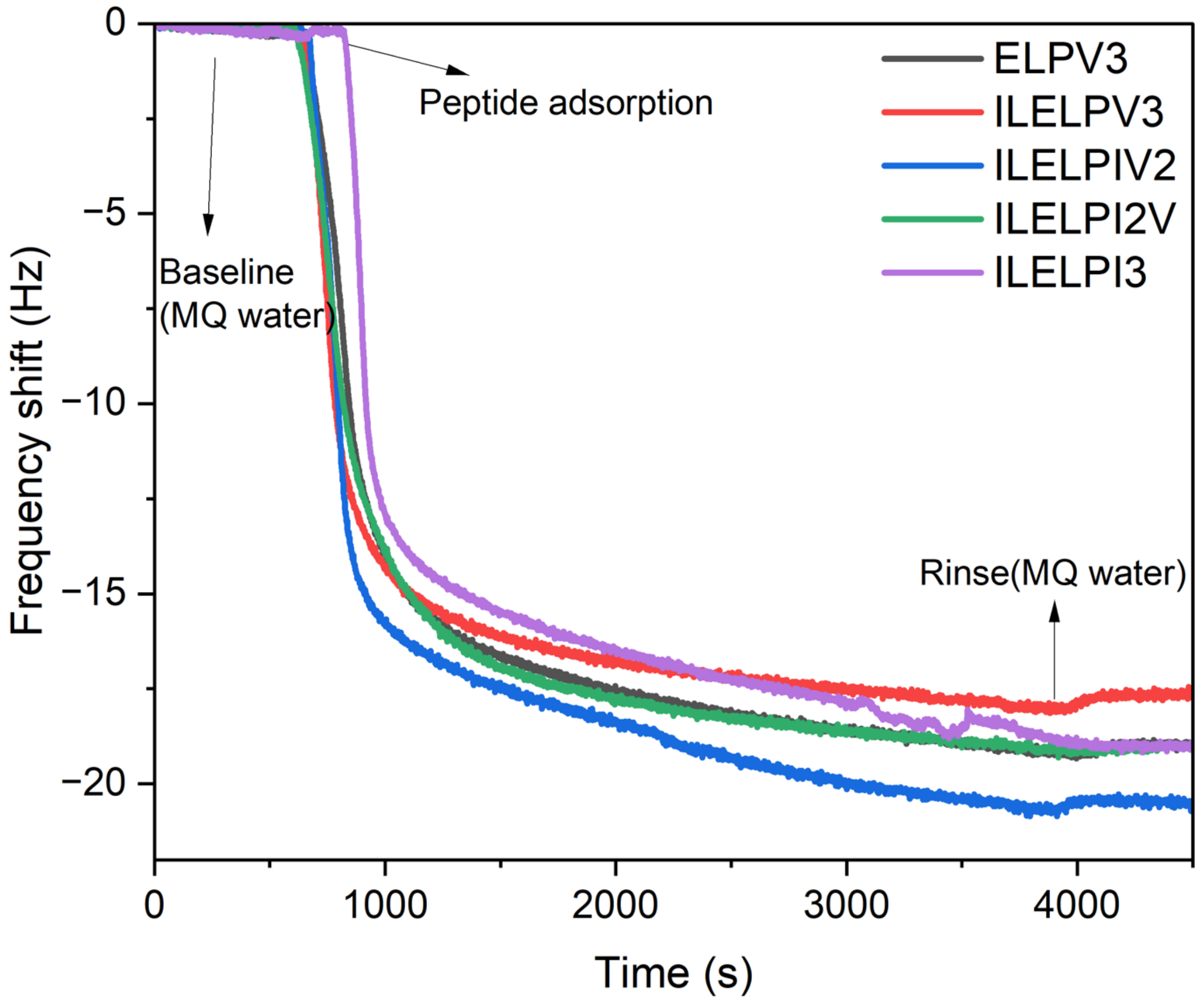


Supplementary Figure 4. QCM-D plots showing frequency shift data vs. time for 10 ppm of designed ELPs as they adsorb to gold-coated quartz crystal sensors. The frequency shift data show similar mass loading for all ELPs. Each experiment was performed once.

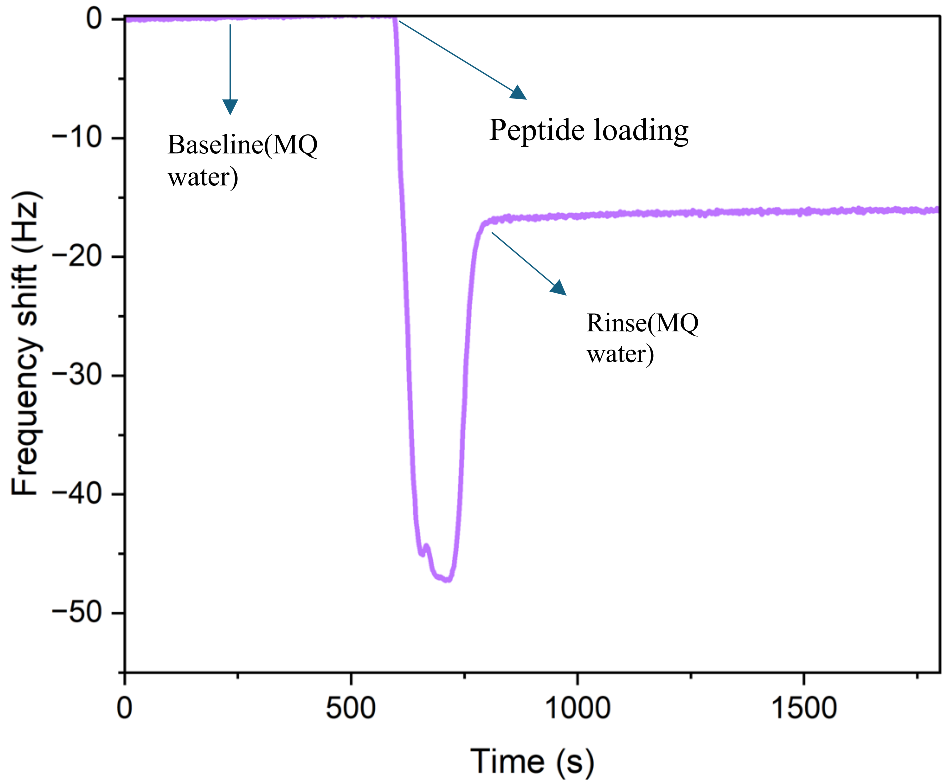


Supplementary Figure 5. QCM-D plots showing frequency shift data vs. time for 1 mg/mL solution of ILELPI3 on a gold-coated quartz crystal sensor. The frequency shift shows a similar loaded mass after rinse compared to the 10 ppm solution of ILELPI3 (Fig. S4). This experiment was performed once.

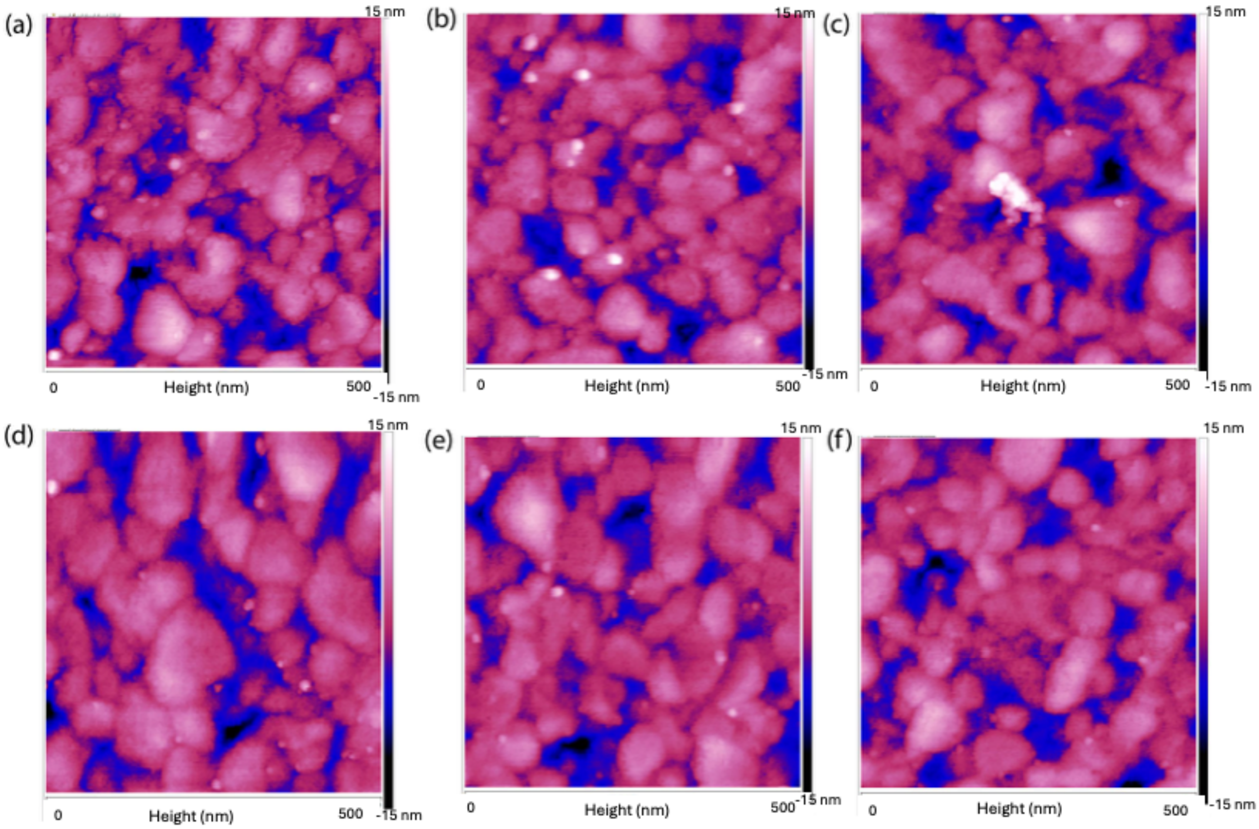


Supplementary Figure 6. AFM measurements show topography data for a) ELPV3 b) ILELPV3 c) ILELPIV2 d) ILELPI2V e) ILELPI3 and f) bare gold. This shows similar morphology for all designed peptides with different hydrophilicity. One image was taken on one single prepared sample.

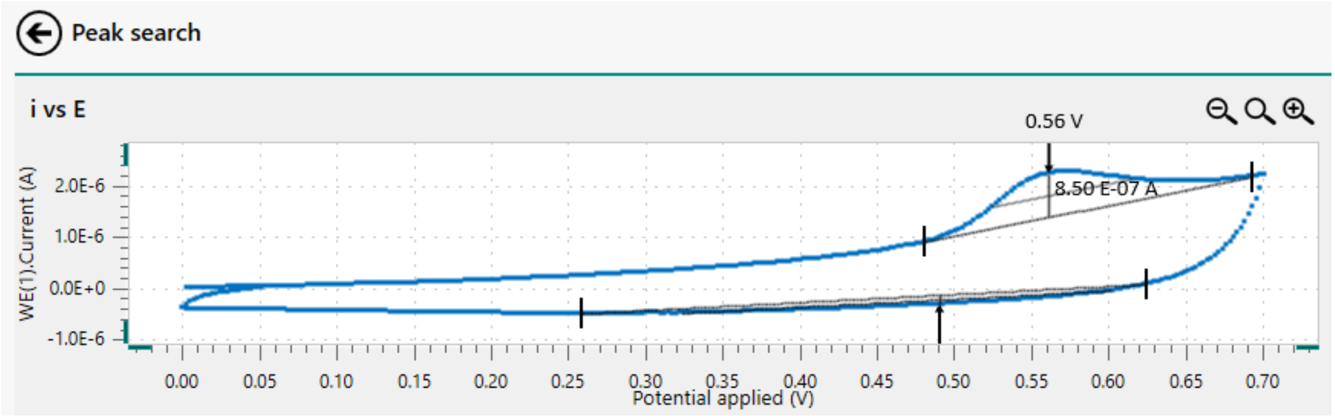


Supplementary Figure 7. Cyclic voltammetry (CV) plot done on gold SPE immobilized with ILELPV3. The electrolyte solution was 0.05 M NaCl and 0.1 M potassium phosphate buffer solution, performed from 0 to 0.7 V vs. pseudo reference electrode at a scan rate of 50 mV/s. Peak height was determined by Nova software. An example screenshot of peak and peak height identification is shown for convenience.

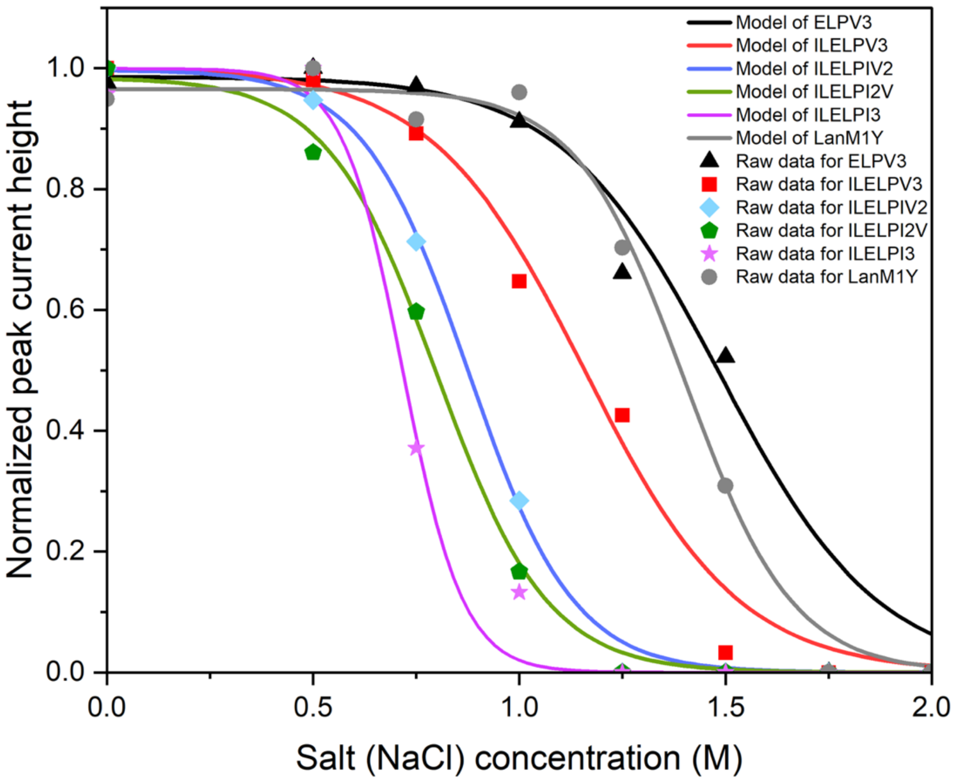


Supplementary Figure 8. Normalized peak current height versus salt (NaCl) concentration obtained by CV for ELPV3 (black triangles show data average, black line shows model), ILELPV3 (red squares show data average, red line shows model), ILELPIV2 (blue rhombus show data average, blue line shows model), ILELPI2V (green parallelograms show data average, green line shows model), ILELPI3 (purple stars show data average, purple line shows model), and LanM1Y (gray circles show data average, gray line shows model). Supplementary Table1 has the exact replicates per data point shown in the plot (each repeat was done on a single sensor and modeled via Eq. 1). CV was performed between 0 to 0.7 V vs. silver pseudo reference electrode with a scan rate of 50 mV/s.

Supplementary Table 1. Exact replicates per data point are shown in the Supplementary Figure 8

| | No. of repeats per each data points | | | | |
|---|---|---|---|---|---|
| Salt concentration | ELPV3 | ILELPIV2 | ILELPI2V | ILELPI3 | LanM1Y |
| 0 | 3 | 7 | 5 | 5 | 1 |
| 0.5 | 3 | 5 | 3 | 3 | 1 |
| 0.75 | 3 | 3 | 5 | 4 | 1 |
| 1 | 3 | 3 | 6 | 6 | 1 |
| 1.25 | 3 | 7 | 6 | 6 | 1 |
| 1.5 | 3 | 7 | 5 | 5 | 1 |
| 1.75 | 3 | 3 | 3 | 3 | 1 |
| 2 | 3 | 3 | 3 | 3 | 1 |

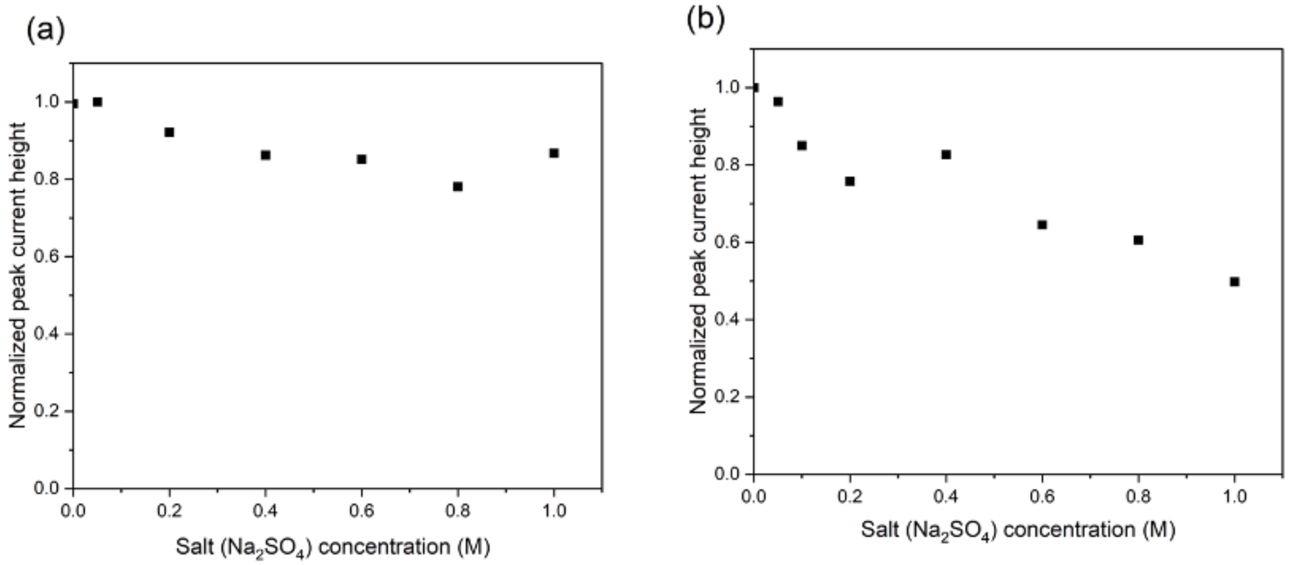


Supplementary Figure 9. Normalized peak current height versus salt ($Na_2SO_4$) concentration. a) ELPV3 b) ILELPI3. The normalized peak current height of CV data is a single repeat (each data point has been done once on a single sensor). CV was performed between 0 to 0.5 V vs. a silver pseudo-reference electrode with a scan rate of 50 mV/s.

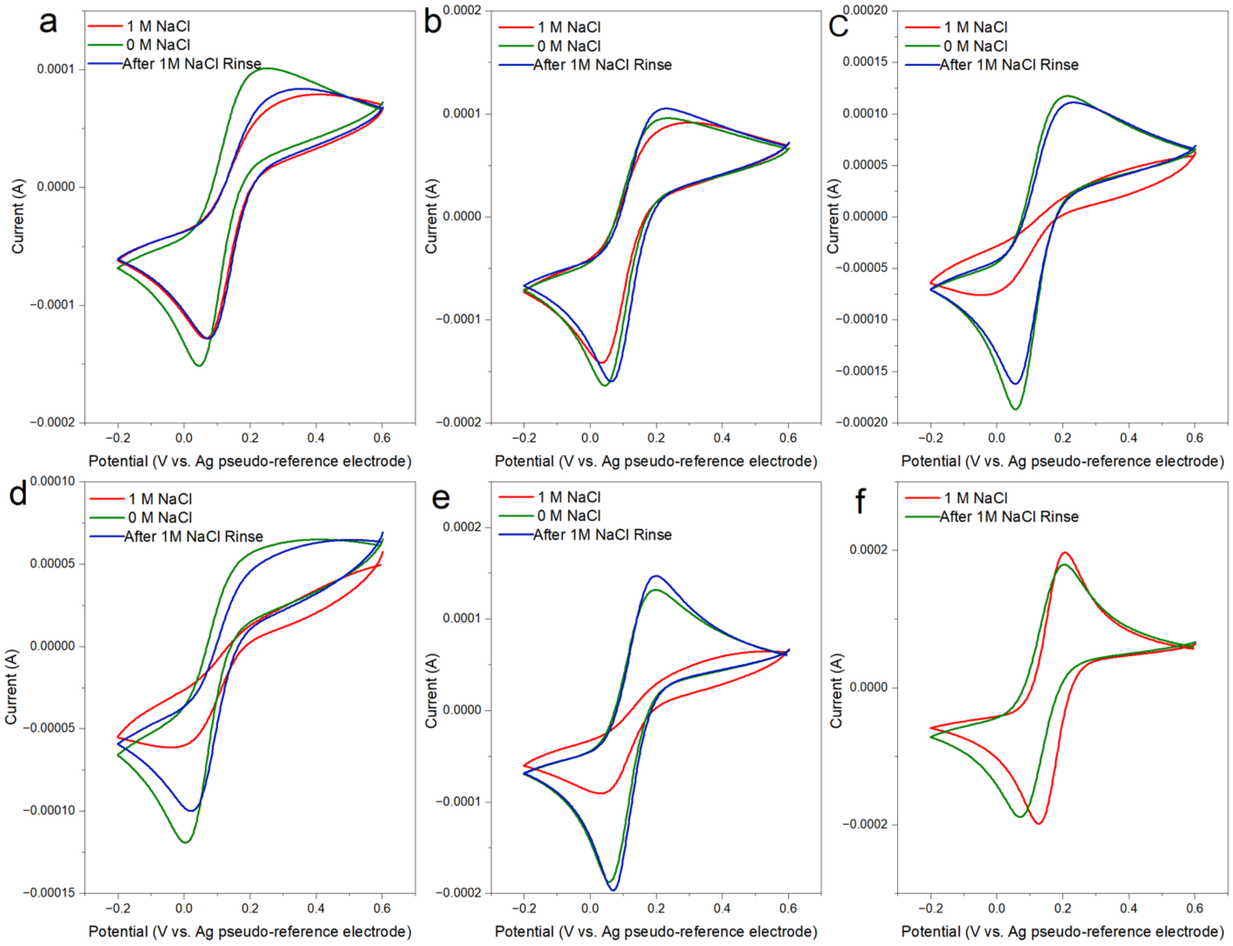


Supplementary Figure 10. Reversibility plots showing the reversible behavior of ELPs (1 mg/mL of peptide solutions incubated on the working electrode of SPEs) a) ELPV3 b) ILELPV3 c) ILELPIV2 d) ILELPI2V e) ILELPI3 and f) bare gold. Electrodes were exposed to a high salt environment (1 M NaCl solution) and a CV was performed (red curve), then the electrodes were rinsed and CV performed a second time (blue curve). Another peptide-functionalized sensor was exposed to the iron ferricyanide redox probe without salt (green curve) for comparison. Cyclic voltammetry was done within -0.2 to 0.6 V vs. pseudo reference electrode, scan rate of 100 mV/s with ferricyanide/ferrocyanide redox couple buffer solution of 0 M salt concentration and 1 M salt concentration. (ELPV3 and ILELPV3 are only dissolved in MQ water, not MQ and <10% DMSO for this specific experiment). Differences between red and blue indicate a transition has occurred, and similarity between blue and green indicates reversibility. Each experiment has been done once on single sensor.

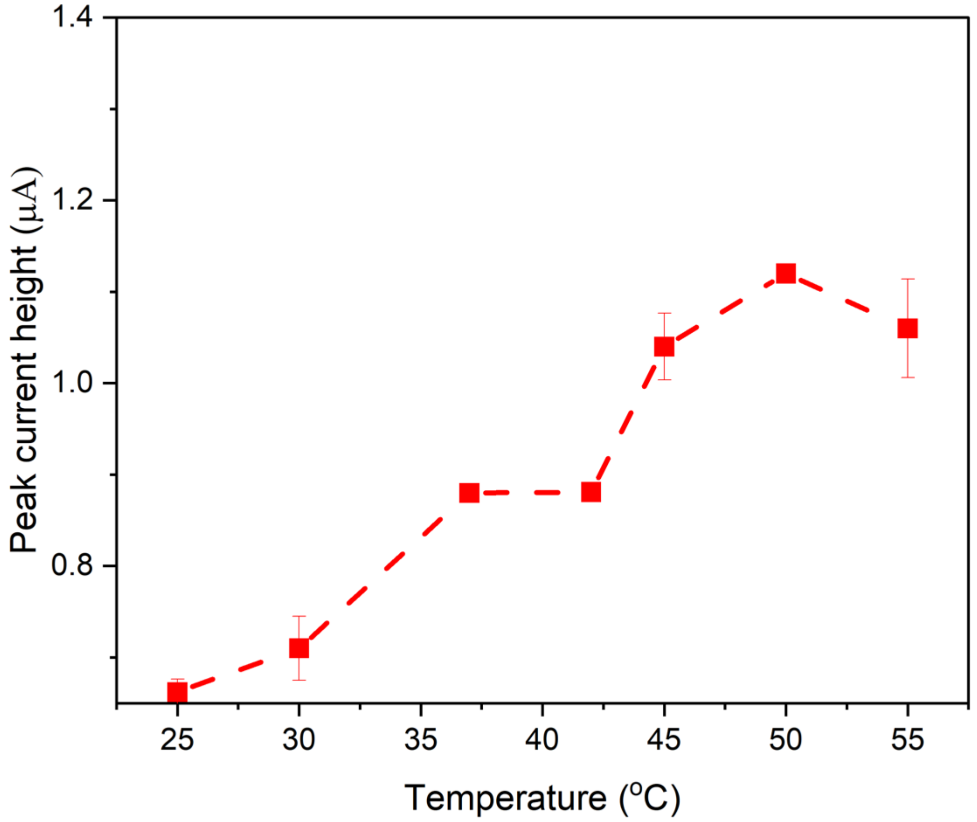


Supplementary Figure 11. Peak current height versus temperature plot of electrodes functionalized with ILELPV3. The peak height of CV data was averaged between 2 repeats (each repeat was done on a single sensor). Cyclic voltammetry was performed from 0 to 0.7 V vs. pseudo-silver reference electrode with a scan rate of 50 mV/s. Error bars represent ± standard errors. Segments are to connect the reader's eye to the next consecutive temperature. Two repeats were performed for each data point.

Supplementary Table 2A. ANOVA results at α = 0.05 for the data presented in Figure 3 (peak current height at different temperatures). For all peptides except ILELPI2V and ILELPI3, the data met normality and equal-variance assumptions and were analyzed using one-way ANOVA followed by Tukey's post hoc test. ILELPI2V did not meet normality assumptions and was analyzed using the Kruskal–Wallis test followed by Dunn's post hoc test. ILELPI3 exhibited unequal variances and was analyzed using Welch's one-way ANOVA followed by a Games–Howell post hoc test.

| ELPV3 | | | | | |
|---|---|---|---|---|---|
| **Source** | DF | Adj SS | Adj MS | F-value | P-value |
| **ELPV3** | 6 | 0.70 | 0.12 | 14.15 | 0.000 |
| **Error** | 14 | 0.12 | 0.01 | | |
| **Total** | 20 | 0.82 | | | |

| ELPV3 | | | | |
|---|---|---|---|---|
| **Temperature** | N | Mean | StDEv | 95% CI |
| **25** | 3 | 0.89 | 0.028 | (0.078, 1.002) |
| **32** | 3 | 1.20 | 0.057 | (1.084, 1.309) |
| **35** | 3 | 1.21 | 0.071 | (1.094, 1.319) |
| **40** | 3 | 1.27 | 0.068 | (1.154, 1.379) |
| **45** | 3 | 1.39 | 0.080 | (1.274, 1.500) |
| **50** | 3 | 1.41 | 0.171 | (1.297, 1.523) |
| **55** | 3 | 1.49 | 0.093 | (1.374, 1.599) |

| ILELPIV2 | | | | | |
|---|---|---|---|---|---|
| **Source** | DF | Adj SS | Adj MS | F-value | P-value |
| **ILELPIV2** | 6 | 0.79 | 0.13 | 20.95 | 0.000 |
| **Error** | 29 | 0.18 | 0.006 | | |
| **Total** | 35 | 0.98 | | | |

| ILELPIV2 | | | | |
|---|---|---|---|---|
| **Temperature** | N | Mean | StDEv | 95% CI |
| **25** | 5 | 0.73 | 0.06 | (0.66, 0.80) |
| **30** | 6 | 0.88 | 0.07 | (0.81, 0.95) |
| **35** | 5 | 0.84 | 0.02 | (0.76, 0.91) |
| **40** | 5 | 0.89 | 0.05 | (0.82, 0.97) |
| **45** | 4 | 1.17 | 0.12 | (1.08, 1.25) |
| **50** | 5 | 1.15 | 0.13 | (1.08, 1.22) |
| **55** | 6 | 1.05 | 0.07 | (0.99, 1.12) |

| ILELPI2V | | | |
|---|---|---|---|
| **Source** | DF | H-value | P-value |
| **ILELPIV2** | 6 | 27.59 | 0.000 |
| **Adjusted for Ties** | 6 | 27.59 | 0.000 |

Supplementary Table 2A continued

| ILELPI2V | | | |
|---|---|---|---|
| **Temperature** | N | Median | 95% CI |
| **25** | 6 | 0.74 | (0.64, 0.87) |
| **30** | 8 | 0.78 | (0.76, 0.96) |
| **35** | 7 | 0.79 | (0.77, 0.99) |
| **40** | 10 | 0.95 | (0.91, 1.09) |
| **45** | 10 | 1.10 | (1.03, 1.21) |
| **50** | 8 | 1.11 | (0.97, 1.18) |
| **55** | 9 | 1.00 | (1.01, 1.20) |

| ILELPI3 | | | | |
|---|---|---|---|---|
| **Source** | DF Numerator | DF Denominator | F-value | P-value |
| **ILELPI3** | 6 | 22.01 | 45.37 | 0.00 |

| ILELPI3 | | | | |
|---|---|---|---|---|
| **Temperature** | N | Mean | StDEv | 95% CI |
| **25** | 8 | 0.69 | 0.05 | (0.65, 0.73) |
| **30** | 11 | 0.82 | 0.16 | (0.72, 0.93) |
| **35** | 7 | 0.79 | 0.11 | (0.69, 0.90) |
| **40** | 8 | 1.06 | 0.12 | (0.96, 1.16) |
| **45** | 10 | 1.08 | 0.06 | (1.04, 1.12) |
| **50** | 9 | 1.16 | 0.11 | (1.07, 1.24) |
| **55** | 7 | 1.11 | 0.16 | (0.96, 1.25) |

Supplementary Table 2B. Statistical groupings and results for post hoc tests at $\alpha = 0.05$ (95% confidence). Groups that do not share a letter are statistically different. For all peptides except ILELPI2V and ILELPI3, the data met normality and equal-variance assumptions and were analyzed using one-way ANOVA followed by Tukey's post hoc test. ILELPI2V did not meet normality assumptions and was analyzed using the Kruskal–Wallis test followed by Dunn's post hoc test. ILELPI3 exhibited unequal variances and was analyzed using Welch's one-way ANOVA followed by a Games–Howell post hoc test.

| ELPV3 | | | | |
|---|---|---|---|---|
| **temperature** | Number of repeats | grouping | | |
| **25** | 3 | A | | |
| **32** | 3 | | B | |
| **35** | 3 | | B | |
| **40** | 3 | | B | C |
| **45** | 3 | | B | C |
| **50** | 3 | | B | C |
| **55** | 3 | | | C |

| ILELPIV2 | | | | |
|---|---|---|---|---|
| **temperature** | Number of repeats | grouping | | |
| **25** | 5 | A | | |
| **30** | 6 | A | B | |
| **35** | 5 | A | B | |
| **40** | 5 | | B | |
| **45** | 4 | | | C |
| **50** | 5 | | | C |
| **55** | 6 | | | C |

| ILELPI2V (significant differences shown instead of groupings) | | |
|---|---|---|
| Groups | Z vs. Critical value | P-value |
| 25 vs. 45 | 3.99455 >= 2.593 | 0.0001 |
| 25 vs. 50 | 3.49057 >= 2.593 | 0.0005 |
| 30 vs. 45 | 3.23033 >= 2.593 | 0.0012 |
| 25 vs. 55 | 3.15854 >= 2.593 | 0.0016 |
| 35 vs. 45 | 2.90974 >= 2.593 | 0.0036 |
| 30 vs. 50 | 2.70925 >= 2.593 | 0.0067 |

| ILELPI3 | | | |
|---|---|---|---|
| **temperature** | Number of repeats | grouping | |
| **25** | 8 | A | |
| **30** | 11 | A | |
| **35** | 7 | A | |
| **40** | 8 | | B |
| **45** | 10 | | B |
| **50** | 9 | | B |
| **55** | 7 | | B |

Supplementary Table 3. Number of repeats for each data point for Figure 3

| | No. of repeats per each data points | | | |
|---|---|---|---|---|
| Temperature(ºC) | ELPV3 | ILELPIV2 | ILELPI2V | ILELPI3 |
| 25 | 3 | 5 | 6 | 8 |
| 30 | 3 | 6 | 8 | 11 |
| 35 | 3 | 5 | 7 | 7 |
| 40 | 3 | 5 | 10 | 8 |
| 45 | 3 | 4 | 10 | 10 |
| 50 | 3 | 5 | 8 | 9 |
| 55 | 3 | 6 | 9 | 7 |

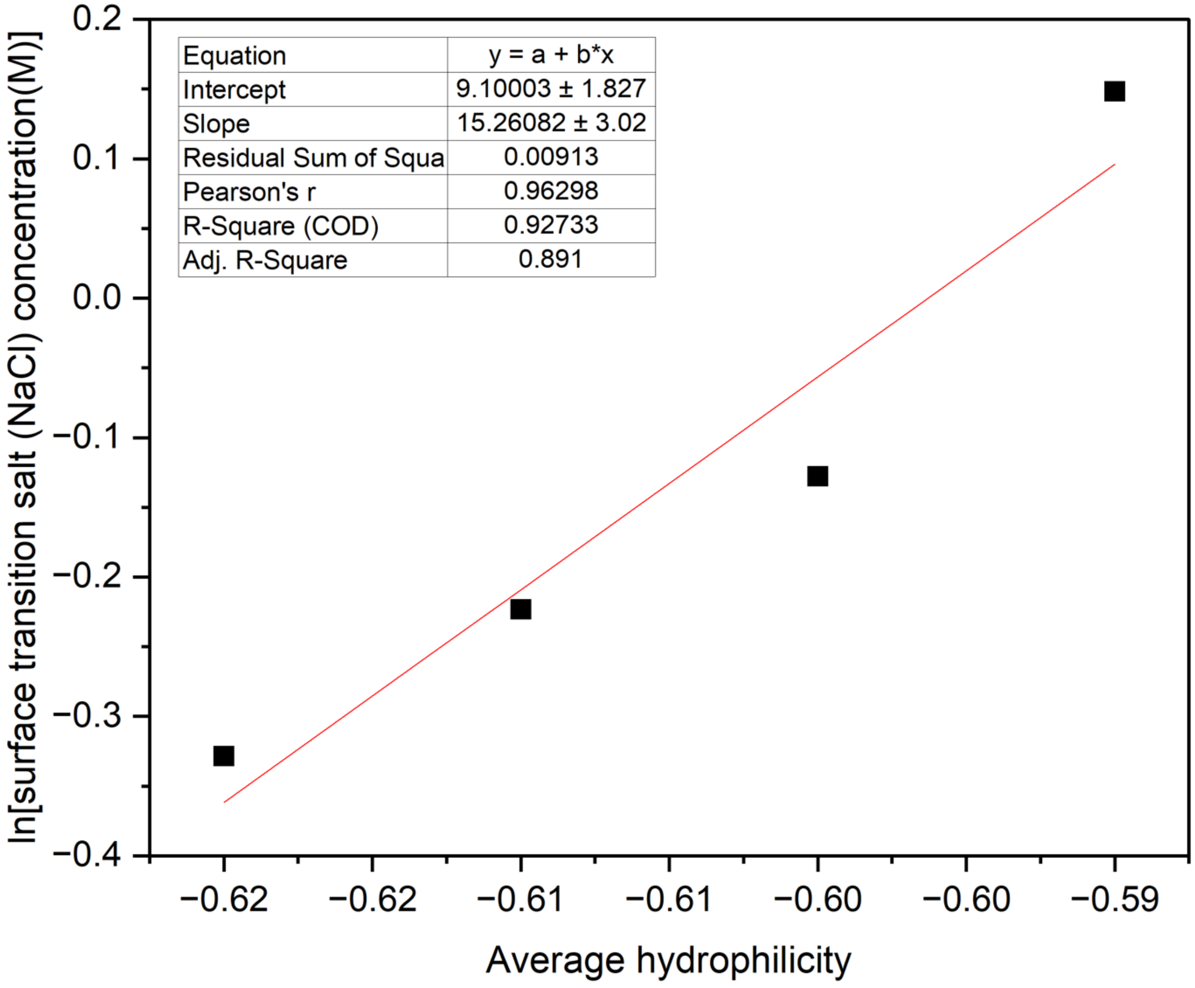


Supplementary Figure 12. Linear fit of the natural logarithm of the NaCl concentration at which ELPs undergo transition vs. the average hydrophilicity of peptides calculated in Table 1. The red line represents the linear regression model shown in Equation 2.

Supplementary Table 4. ANOVA results for the data presented in Figure 4

| Source | DF | Adj SS | Adj MS | F-value | P-value |
|---|---|---|---|---|---|
| **Regression** | 1 | 0.12 | 0.12 | 25.52 | 0.037 |
| **Error** | 2 | 0.009 | 0.005 | | |
| **Total** | 3 | 0.13 | | | |

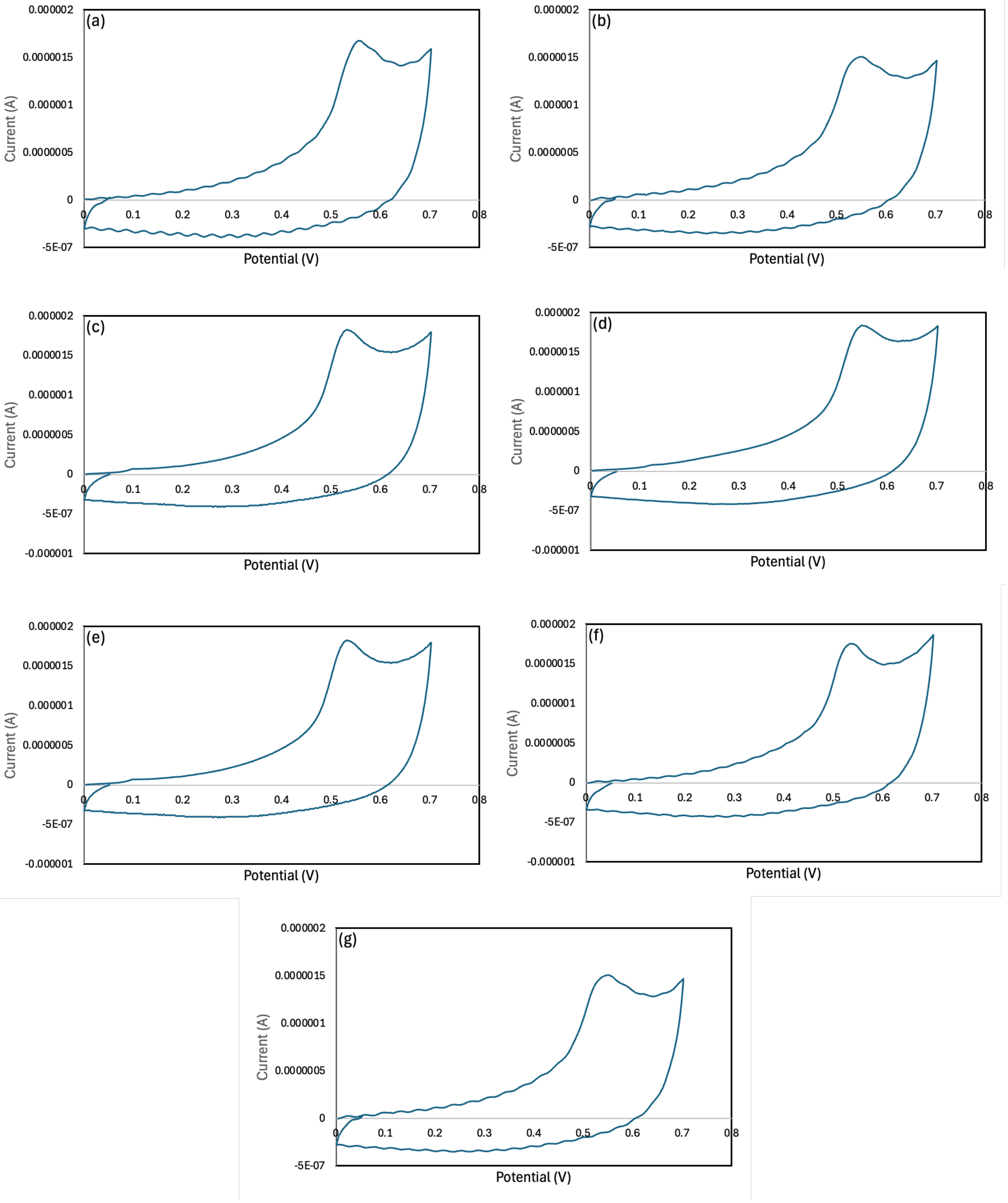


Supplementary Figure 13. An example of raw CV data used in Figure 3 for ILELPI3 at a) 25 °C b) 30 °C c) 35 °C d) 40 °C e) 45 °C f) 50 °C g) 55 °C.

Supplementary equations:

**Immobilized ELP Surface Concentration Calculations**

$\Gamma$= hydrated mass loading measured by QCM-D (ng·cm$^{-2}$)

$A_s$= sensor area (cm$^2$)

$f_d$= dry-mass fraction of the layer (dimensionless; here $f_d = 0.4$)

$h$= film thickness measured by QCM-D (nm)

$m_d$= estimated dry peptide mass on the sensor (ng)

$V$= film volume (cm$^3$)

$C_m$= estimated peptide mass concentration (mg·mL$^{-1}$)

Dry Peptide Mass:

$m_d = f_d \, \Gamma \, A_s$ Supplementary Eq. 1

$\Gamma = 300$ng·cm$^{-2}$, $A_s = 0.95$cm$^2$, $f_d = 0.4$

$m_d = 0.4 \times 300 \times 0.95 = 108$ ng

$V = A_s h \, (10^{-7})$ Supplementary Eq. 2

$A_s = 0.95 \text{ cm}^2, h = 3$ nm:

$V = 0.95 \times 3 \times 10^{-7} = 2.85 \times 10^{-7}\text{cm}^3$

Surface Concentration Based on QCM-D Results:

$C_m$=$m_d \times A_s \times h$ Supplementary Eq. 3

108×10$^{-3}$ug/2.85 ×10$^{-7}$ cm$^3$=3.8×10$^5$ug/cm$^3$=380 mg mL$^{-1}$

Experimental Molecular Density (molecules per unit area) by QCM-D Results:

$M_w$= molecular weight (g·mol$^{-1}$)

$N_A$= Avogadro's number $= 6.022 \times 10^{23}$mol$^{-1}$

$N$= number of peptide molecules on the sensor

$\sigma$= molecular surface density (molecules·cm$^{-2}$)

$$N = \left(\frac{m_d \times 10^{-9}}{M_w}\right) N_A \quad \text{Supplementary Eq. 4}$$

$m_d = 108$ ng and $M_w = 2221.26$ g·mol$^{-1}$:

$$N = (\frac{108 \times 10^{-9}}{2221.26})(6.022 \times 10^{23}) = 2.93 \times 10^{13} \text{ molecules}$$

$$\sigma = \frac{N}{A_s} \quad \text{Supplementary Eq. 5}$$

$$\sigma = \frac{2.93 \times 10^{13}}{0.95} = 3.08 \times 10^{13} \text{ molecules\textbackslash cm}^{-2}$$

Theoretical Molecular Density Calculations Based on Counter Length:

Note: this represents a back-of-the-envelope minimum density required for overlap to occur

$n$= number of residues

b=Contour length per residue (nm·residue$^{-1}$)

L= contour length (nm)

$r_h$= effective hemisphere radius (nm)

$A_{c,h}$= projected area per chain (nm$^2$)

$\sigma_h^*$= minimum overlap grafting density (molecules·nm$^{-2}$)

$$L = nb \quad \text{Supplementary Eq. 6}$$

$n = 25$ and $b = 0.36$ nm/residue:

$$L = 25 \times 0.36 = 9 \text{ nm}$$

$$r_h = \text{L} \quad \text{Supplementary Eq. 7}$$

$L = 9$ nm, $r_h = 9$ nm

$$A_{c,h} = \pi r_h^2 = \pi L^2$$ Supplementary Eq. 8

$$A_{c,h} = \pi(9 \text{ nm})^2 = \pi \times 81 \approx 254\ nm^2$$

Chains begin to interact and overlap when the grafting distance is smaller than the chain size

$$\sigma_h^* = \frac{1}{A_{c,h}}$$ Supplementary Eq. 9

$$\sigma_h^* = \frac{1}{254} = 3.94 \times 10^{-3} \text{ molecules}/nm^2 = 3.94 \times 10^{11} \text{ molecules}/cm^2$$

Supplementary Note 2:

The value of 3 nm thickness by QCM-D to fully extended 9 nm reflects the potential variance in peptide orientation.

Supplementary Note 3: Based on our QCM-D measurements (Supplementary Eq.1–3), the surface-bound ELP concentration after accounting for hydration shell removal is estimated to be on the order of ~380 mg $mL^{-1}$, corresponding to several hundred-fold higher local concentration than that used in solution turbidity measurements. Experimental molecular density is estimated to be $3.8\times10^{13}$ molecules/$cm^2$ (Supplementary Eq.4-5), assuming an averaged of molecular weight for designed ELPs is (2221.26 g/mol). To assess whether such surface concentrations are sufficient to promote interchain interactions, we estimated the minimum molecular density before overlap occurred assuming a half-sphere geometry with contour length of ~9 nm (estimate assumes 0.36 nm (3.6 Å) per amino acid residue[61], a commonly used value for a fully extended polypeptide backbone (Supplementary Eq.6)). This corresponds to a surface density of about 0.004 molecules per $nm^2$ ($\approx 4 \times 10^{11}$ molecules per $cm^2$) (Supplementary Eq.7-9) where chains begin to overlap, which is lower than experimental molecular density. Given the short length and sequence-specific conformational behavior of ELPs, these geometric estimates should be viewed as qualitative rather than quantitative descriptors of intermolecular interactions at the surface.
