# Supplementary material for: Using cyclic voltammetry to probe the conformational transition of short elastin-like peptides
Source: Commun Chem. 2026 Apr 1;9:259. doi: 10.1038/s42004-026-01987-8 (PMC13421663; doi:10.1038/s42004-026-01987-8)
Supplement: Supplementary file 2 — Reporting Checklist For Life Sciences Articles [file 42004_2026_1987_MOESM2_ESM.pdf]

Corresponding Author Name: Julie Renner

Manuscript Number: COMMSCHEM-25-0855A

Reporting Checklist For Life Sciences Articles

This checklist is used to ensure good reporting standards and to improve the reproducibility of published results. For more information, please read Reporting Life Sciences Research.

Figure legends

Each figure legend should contain, for each panel where they are relevant:

- the **exact sample size (*n*)** for each experimental group/condition, given as a number, not a range;
- a **description of the sample collection** allowing the reader to understand whether the samples represent **technical or biological replicates** (including how many animals, litters, cultures, etc.);
- a **statement of how many times the experiment shown was replicated in the laboratory**;
- **definitions of statistical methods and measures**:
  - very common tests, such as *t*-test, simple χ^2 tests, Wilcoxon and Mann-Whitney tests, can be unambiguously identified by name only, but more complex techniques should be described in the methods section;
  - are tests one-sided or two-sided?
  - are there adjustments for multiple comparisons?
  - **statistical test results**, e.g., ***P* values**;
  - definition of '**center values**' as **median or average**;
  - definition of **error bars as s.d. or s.e.m.**

Any descriptions too long for the figure legend should be included in the methods section.

Please ensure that the answers to the following questions are reported in the manuscript itself. We encourage you to include a specific subsection in the methods section for statistics, reagents and animal models. Below, provide the page number(s) or figure legend(s) where the information can be located.

Statistics and general methods

	Reported on page(s) or figure legend(s):
1. How was the sample size chosen to ensure adequate power to detect a pre-specified effect size?	Statistical Analysis Section
For animal studies, include a statement about sample size estimate even if no statistical methods were used.	N/A
2. Describe inclusion/exclusion criteria if samples or animals were excluded from the analysis. Were the criteria pre-established?	N/A
3. If a method of randomization was used to determine how samples/animals were allocated to experimental groups and processed, describe it.	N/A
For animal studies, include a statement about randomization even if no randomization was used.	N/A
4. If the investigator was blinded to the group allocation during the experiment and/or when assessing the outcome, state the extent of blinding.	N/A
For animal studies, include a statement about blinding even if no blinding was done.	N/A
5. For every figure, are statistical tests justified as appropriate?	Statistical Analysis Section
Do the data meet the assumptions of the tests (e.g., normal distribution)?	Statistical Analysis Section
Is there an estimate of variation within each group of data? Is the variance similar between the groups that are being statistically compared?	Statistical Analysis Section

(Continues on following page)

Reagents

Reported on page(s) or figure legend(s):

6. To show that antibodies were profiled for use in the system under study (assay and species), provide a citation, catalog number and/or clone number, supplementary information or reference to an antibody validation profile (e.g., Antibodypedia, 1DegreeBio).

N/A

7. Identify the source of cell lines and report if they were recently authenticated (e.g., by STR profiling) and tested for mycoplasma contamination.

N/A

Animal models

Reported on page(s) or figure legend(s):

8. Report species, strain, sex and age of animals.

N/A

9. For experiments involving live vertebrates, include a statement of compliance with ethical regulations and identify the committee(s) approving the experiments.

N/A

10. We recommend consulting the ARRIVE guidelines (*PLoS Biol.* **8**(6), e1000412, 2010) to ensure that other relevant aspects of animal studies are adequately reported.

Human subjects

Reported on page(s) or figure legend(s):

11. Identify the committee(s) approving the study protocol.

N/A

12. Include a statement confirming that informed consent was obtained from all subjects.

N/A

13. For publication of patient photos, include a statement confirming that consent to publish was obtained.

N/A

14. Report the clinical trial registration number (at ClinicalTrials.gov or equivalent).

N/A

15. For phase II and III randomized controlled trials, please refer to the CONSORT statement and submit the CONSORT checklist with your submission.

16. For tumor marker prognostic studies, we recommend that you follow the REMARK reporting guidelines.

Data deposition

17. Provide accession codes for deposited data.

Reported on page(s) or figure legend(s):

Data deposition in a public repository is mandatory for:

a. Protein, DNA and RNA sequences
b. Macromolecular structures
c. Crystallographic data for small molecules
d. Microarray data

Deposition is strongly recommended for many other datasets for which structured public repositories exist; more details on our data policy are available here. We encourage the provision of other source data in supplementary information or in unstructured repositories such as Figshare and Dryad.

N/A

18. Is computer source code provided with the paper or deposited in a public repository? If so, indicate how it can be obtained.

N/A
